# Novel Isoxazole-Based Antifungal Drug Candidates

**DOI:** 10.3390/ijms252413618

**Published:** 2024-12-19

**Authors:** Urszula Bąchor, Malwina Brożyna, Adam Junka, Mateusz Ramires Chmielarz, Damian Gorczyca, Marcin Mączyński

**Affiliations:** 1 Department of Organic Chemistry and Drug Technology, Faculty of Pharmacy, Wroclaw Medical University, 50-556 Wroclaw, Poland; urszula.bachor@umw.edu.pl; 2 Unique Application Model Laboratory, Department of Pharmaceutical Microbiology and Parasitology, Faculty of Pharmacy, Wroclaw Medical University, 50-556 Wroclaw, Poland; 3 Department of Clinical Microbiology, Faculty of Medicine, Wroclaw Medical University, Chalubinskiego 4, 50-368 Wroclaw, Poland; 4 Faculty of Medicine, Lazarski University, 02-662 Warszawa, Poland

**Keywords:** isoxazoles, anti-*Candida*, biofilm

## Abstract

Microbiological communities have a significant impact on health and disease. *Candida* are ubiquitous fungal pathogens that colonize the mucosal surfaces of the genital, urinary, respiratory, and gastrointestinal tracts, as well as the oral cavity. If the immune system is inadequate, then *Candida* infections may pose a significant threat. Due to the limited number of clinically approved drugs for the treatment of *Candida albicans*-based infections and the rapid emergence of resistance to the existing antifungals, a novel series of isoxazole-based derivatives was synthesized and evaluated in vitro for their anti-*Candida* potential. Two compounds, **PUB14** and **PUB17**, displayed selective antifungal activity without negatively affecting beneficial microbiota, such as *Lactobacillus* sp., at the same time. Moreover, these compounds exhibited significantly lower cytotoxicity in comparison to conventionally applied local antimicrobial (octenidine dihydrochloride), indicating their potential for safe and effective clinical application in conditions such as vulvovaginal candidiasis. The selective antifungal activity of **PUB14** and **PUB17** against *C. albicans*, coupled with its absence of antibacterial effects and minimal cytotoxicity towards HeLa cells, suggests a targeted mechanism of action that warrants further investigation. Consideration of the need to search for new antifungal agents and the discovery of an antifungal potential drug that does not inhibit lactobacilli growth could be a potential strategy to prevent and combat vulvovaginal candidiasis. This striking capacity to eradicate biofilm formed by *Candida* reveals a new approach to eradicating biofilms and sheds light on isoxazole-based derivatives as promising anti-biofilm drugs.

## 1. Introduction

Research into microbial biofilms has gained increasing momentum over the past two decades, at least. It is now accepted that biofilms are the preferred and likely natural state of growth for most microorganisms, including these involved in development of pathogenic entities among animals and humans. Nonetheless, an almost universal characteristic of biofilms is their elevated tolerance towards antimicrobial agents, rendering them exceedingly challenging to combat in clinical settings and a burden to overcome from the perspective of human health. The number of recognized infections caused by biofilm-forming fungal pathogens is still increasing. For instance, *Candida* biofilm formation has been observed on multiple surfaces, including biotic (organs, blood vessels and vaginal mucosa) and abiotic ones (stents, implants and multiple types of catheters) [1].

*Candida albicans* is a yeast fungus that naturally occurs in the microbiota of the human urinary and digestive system, as well as on the skin or upper respiratory tracts. However, mostly due to a decrease in the body’s immunity, *Candida albicans* develop a wide spectrum of so-called opportunistic infections.

Vulvovaginal candidiasis (VVC) is an infectious disease that affects the female genital tract and is caused by *Candida* species [2]. In fact, *Candida* account for approximately 75–88% of all fungal infections in the US. Moreover, about 75% of women suffer from VVC at least once during their lifetime [3].

Both *Lactobacillus* spp. and *Candida* spp. are commensals of the human microbiome. Additionally, *Lactobacilli* have been shown to directly inhibit *C. albicans* growth in vitro by producing lactic acid. Several studies have associated VVC with a low number of lactobacilli in the vagina. Lactic acid is a natural component of vaginal secretions, and is mainly generated by vaginal lactobacilli [4]. In healthy women, the predominant vaginal microbiota, such as *Lactobacillus crispatus*, inhibits pathogen growth through the production of anti-microbial compounds such as lactic acid and H_2_O_2_.

Treatment options for VVC comprise a variety of antifungal agents, containing different heterocyclic cores, e.g., triazole (fluconazole) or imidazole, such as miconazole or clotrimazole. Clotrimazole is a topical broad-spectrum antifungal agent used for the treatment of a wide variety of dermatophyte infections, especially to treat vulvovaginal candidiasis (VVC) [5].

Ross et al. studied the effects of *C. albicans* infection and clotrimazole treatment on vaginal microbiota in vitro [6]. Following *C. albicans* challenge, the model developed abnormal microbiota. The treatment of the model with clotrimazole resulted in a decrease in *C. albicans*, but also altered other components of the vaginal microbiota, which did not return to appropriate state regarding its composition. The use of clotrimazole is associated with an increased risk of infection or disease due to the disruption of the protective microbiota barrier [7]. Moreover, nowadays, *Candida* sp. is reported to gain resistance to oral azoles that could potentially cause systemic toxicity. Antibiotic resistance, adverse effects and recurrences are still recognized as a significant challenge for clinicians when using clotrimazole to treat VVC.

The increasing demand for new effective drugs and the discovery of new biologically active molecules have resulted in a continuous search for simple and efficient methods for obtaining biological libraries. Therefore, medicinal chemists are particularly interested in synthetic methodologies that allow easy access to large databases of compounds. Considering that, the multidirectional synthesis and acquisition of new functions by compounds with known biological activity enable the creation of a library of structurally diverse molecules with new pharmacologic activities. Among all known pharmaceuticals, a predominant amount of them are small molecules with heterocyclic moieties. Among all known heterocyclic cores, our attention is focused on azoles (oxazoles, pyrazoles, imidazoles, thiazole) derivatives as they show a wide range of biological activity, including anticancer [8], anti-inflammatory and antioxidant [9,10] or antimicrobial activity [11,12,13].

Among them, isoxazole derivatives present a large spectrum of targets and broad biological activities, anticancer [14], immunosuppressive [15] and antiviral [16]. Successful applications of developing isoxazole compounds have resulted in multiple drugs containing this heterocyclic core. It is noteworthy to mention that sulfonamide antibiotics possess distinct heterocyclic moieties, such as the presence of 1,2-oxazole, pyrazole, and 1,3-thiazole rings in sulfamethoxazole, sulfaphenazole, and sulfathiazole, respectively.

Sulfamethoxazole has been approved for the treatment of severe, repeated, or long-lasting urinary tract infections by inhibiting the enzyme dihydropteroate synthetase.

Heterocyclic compounds are the leading structures for synthesis of its numerous derivatives, which have been shown to possess a wide range of pharmacological activities and exhibited a very important role in development of many drugs. Azoles such as imidazole and thiazole are extremely valuable molecules in the fields of organic synthesis and biological activity.

**Figure 1:**
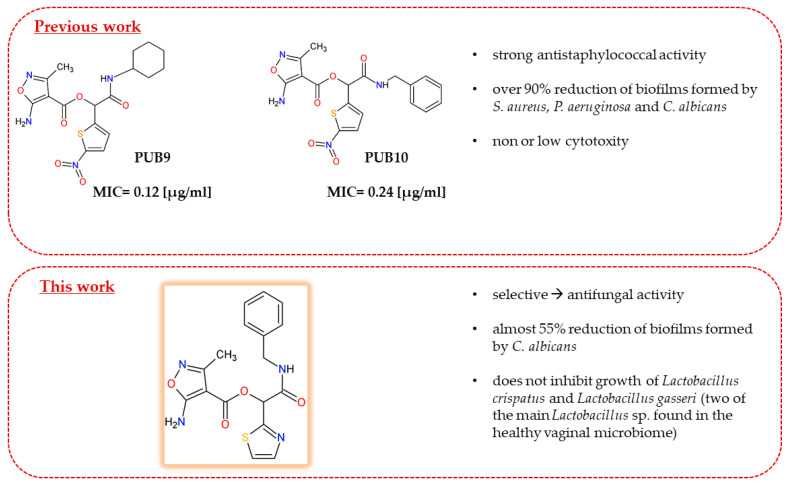
Isoxazole-based derivatives with antimicrobial activity obtained by our team.

Isoxazole/thiophene-based derivative (**PUB9**) obtained by us has been identified as excellent growth inhibitor of *S. aureus* [17]. In this decade, great attention has been paid to developing new heterocyclic-based drug candidates with potential antimicrobial activity. In view of this, many structurally diversified thiazole [18], imidazole (Clotrimazole, Butoconazole, Ketoconazole) [19] or isoxazole [20] derivatives were designed, synthesized and screened for their in vitro and in vivo efficacy to act as antimicrobial agents. Basically, nitrogen/sulfur-containing heterocycles have attracted particular attention in recently published studies due to their valuable biological properties [21,22,23]. In particular, a 1,3-thiazole scaffold seems to be crucial for antimicrobial activity [24], and has been reported many times as an important moiety of numerous antifungal agents [22,25,26,27]. Because previous studies on the biological activities of isoxazole-based heterocyclic system indicate that these compounds exhibit a wide range of antimicrobial properties, it seems reasonable to search in this class of compounds a potential antimicrobial agent. Previously obtained by us new 5-amino-3-methylisoxazole-4-carboxylic acid derivatives exhibiting strong antibacterial properties (Figure 1), prompted us to expect from this class of compounds (containing more than one heterocyclic moiety) a potential therapeutic utility [17]. Following this hypothesis, and consistent with the interest of our research group in isoxazole-based compounds, we decided to synthesize a new series of molecules containing an isoxazole heterocyclic system as a valuable source in the search for potential drugs. Thus, we obtained a new series of isoxazole-based heterocyclic hybrids, and then we scrutinized all compounds due to their antimicrobial properties.

## 2. Results

### 2.1. Chemistry

The main substrate for further synthesis, 5-amino-3-methylisoxazole-4 carboxylic acid, was synthesized according to the procedure described earlier [28,29]. A new series of isoxazole-based α-acyloxyamides was obtained via Passerini multicomponent reaction. The structures of all of the new compounds were determined by ^1^H NMR, ^13^C NMR and MS (for details, see Section 4).

#### General Procedure for Preparation of a Series of Compounds (**PUB11**–**PUB18**)

A novel series of isoxazole-based heterocyclic hybrid compounds (referred to in this work as the **PUB11**–**18** series) were obtained using Passerini three-component reaction.

An amount of 2.1 mmol of 5-amino-3-methylisoxazole-4 carboxylic acid and appropriate aldehyde (2.1 mmol) were dissolved in 5 mL of THF. The reaction mixture was heated (if necessary) to dissolve substrates, then cyclohexyl isocyanide or benzyl isocyanide was added (2.1 mmol). The reaction mixture was heated under reflux for 72 h or 96 h. Upon completion of the reaction, as shown by TLC, it was cooled to room temperature and the formed precipitate was filtered off and dried. The final product was purified by crystallization from THF or methanol, or via flash chromatography; mobile phase: chloroform/methanol (gradient elution).

### 2.2. Synthesis and Structural Characterization

**Figure 2:**
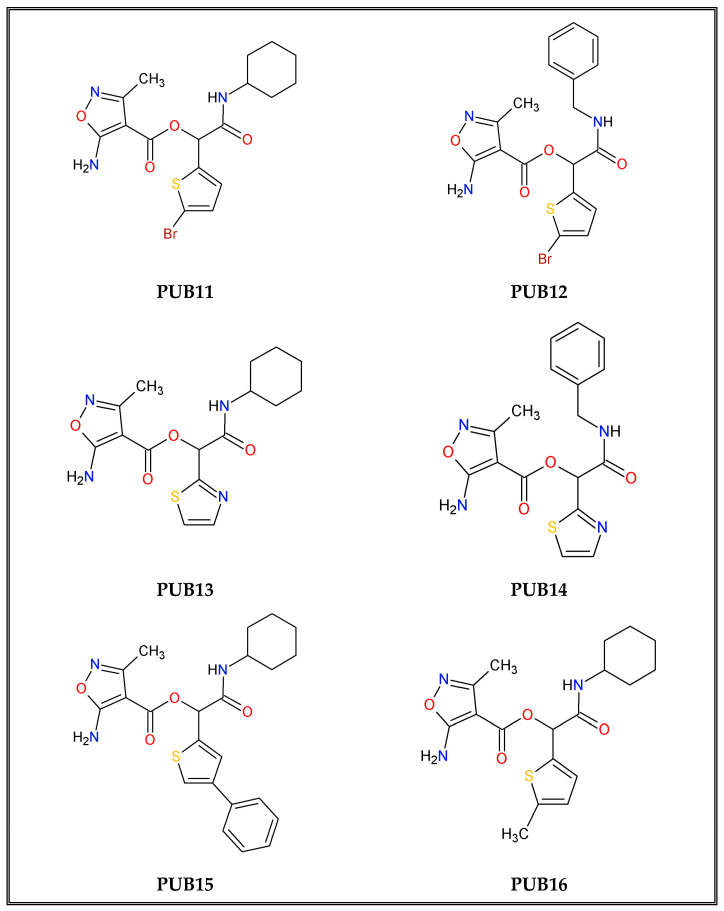

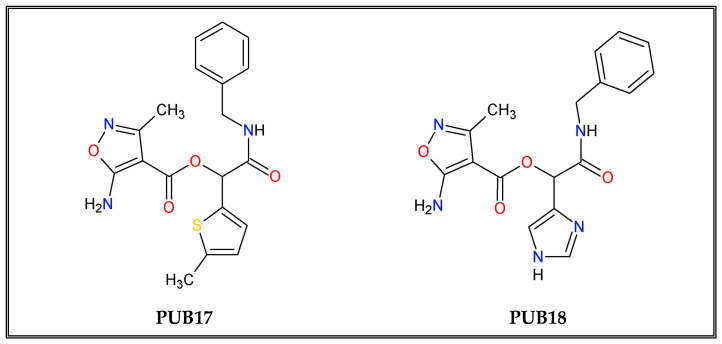
The series of isoxazole-based derivatives **PUB11**–**18**.

Structures of new compounds are shown below in the Figure 2. The Mass Spectrometry (MS) and Nuclear Magnetic Resonance (NMR) spectral analysis of new compounds and their spectra visualization are shown below and in Supplementary Materials (Figure S1–S24), as well as physicochemical properties of compounds **PUB11**–**PUB18** (Table S1).

1-(5-bromothiophen-2-yl)-2-(cyclohexylamino)-2-oxoethyl 5-amino-3-methyl-1,2-oxazole-4-carboxylate (**PUB11**)
7.5% yield; m.p. 205–206 °C, beige solid. ^1^H NMR (600 MHz, DMSO-*d*_6_) δ (ppm): 1.02–1.31 (m, 5H), 1.48–1.78 (m, 5H). 2.21 (s, 3H, CH_3_ group of isoxazole ring), 3.51 (m, 1H), 6.21 (s, 1H), 7.03 (d, *J* = 3.8 Hz, 1H), 7.14 (d, *J* = 3.8 Hz, 1H), 7.85 (bs, 2H, NH_2_ group from isoxazole ring), 8.28 (d, *J* = 7.8 Hz, 1H). ^13^C NMR (151 MHz, DMSO-*d*_6_) δ (ppm): 12.08, 12.34, 24.85, 25.56, 32.42, 32.51, 48.31, 70.51, 84.61, 70.51, 84.61, 112.88, 128.33, 130.28, 140.88, 159.19, 161.46, 166.31, 172.28. ESI-MS: *m*/*z* calculated for formula C_17_H_20_BrN_3_O_4_S [M+H]^+^ 442.043, found [M+H]^+^ 442.041.
2-(benzylamino)-1-(5-bromothiophen-2-yl)-2-oxoethyl 5-amino-3-methyl-1,2-oxazole-4-carboxylate (**PUB12**)
3.2% yield; m.p. 185–186 °C, beige solid. ^1^H NMR (600 MHz, DMSO-*d*_6_) δ (ppm): 2.23 (s, 3H, CH_3_ group of isoxazole ring), 4.22–4.39 (m, 2H), 6.31 (s, 1H), 7.08 (d, *J* = 3.8 Hz, 1H), 7.15–7.32 (m, 5H), 7.89 (bs, 2H, NH_2_ group from isoxazole ring), 8.92 (t, *J* = 6.0 Hz, 1H). ^13^C NMR (151 MHz, DMSO-*d*_6_) δ (ppm): 12.43, 42.57, 70.84, 84.57, 112.97, 127.37, 127.52, 128.75, 130.42, 139.16, 140.12, 159.37, 161.64, 167.64, 172.58. ESI-MS: *m*/*z* calculated for formula C_18_H_16_BrN_3_O_4_S [M+H]^+^ 450.012, found [M+H]^+^ 450.010.
2-(cyclohexylamino)-2-oxo-1-(1,3-thiazol-2-yl)ethyl 5-amino-3-methyl-1,2-oxazole-4-carboxylate (**PUB13**)
11% yield; m.p. 225–227 °C, beige solid. ^1^H NMR (600 MHz, DMSO-*d*_6_) δ (ppm): 1.32–1.06 (m, 5H), 1.79–1.47 (m, 5H), 2.24 (s, 3H, CH_3_ group of isoxazole ring), 3.58–3.47 (m, 1H), 6.34 (s, 1H), 7.80 (d, *J* = 3.2 Hz, 1H), 7.84 (d, *J* = 3.2 Hz, 1H), 7.93 (bs, 2H, NH_2_ group from isoxazole ring), 8.45 (d, *J* = 7.8 Hz, 1H). ^13^C NMR (151 MHz, DMSO-*d*_6_) δ (ppm): 12.32, 24.84, 25.57, 32.40, 32.48, 48.45, 72.35, 84.51, 122.15, 142.88, 159.38, 161.22, 164.91, 165.24, 172.57. ESI-MS: *m*/*z* calculated for formula C_16_H_20_N_4_O_4_S [M+H]^+^ 365.128, found [M+H]^+^ 365.128.
2-(benzylamino)-2-oxo-1-(1,3-thiazol-2-yl)ethyl 5-amino-3-methyl-1,2-oxazole-4-carboxylate (**PUB14**)
18% yield; m.p. 209–210 °C, beige solid. ^1^H NMR (600 MHz, DMSO-*d*_6_) δ (ppm): 2.26 (s, 1H), 4.29 (dd, *J* = 15.4, 5.9 Hz), 4.36 (dd, *J* = 15.3, 6.1 Hz), 6.43 (s, 1H), 7.19–7.24 (m, 3H), 7.27–7.32 (m, 2H), 7.83 (d, *J* = 3.2 Hz, 1H), 7.87 (d, *J* = 3.2 Hz, 1H), 7.96 (s, 2H, NH_2_ group from isoxazole ring), 9.07 (t, *J* = 6.0 Hz, 1H). ^13^C NMR (151 MHz, DMSO-*d*_6_) δ (ppm): 12.41, 42.64, 72.58, 84.49, 122.32, 127.28, 127.45, 128.70, 139.16, 142.95, 159.41, 161.31, 164.52, 166.47, 172.64. ESI-MS: *m*/*z* calculated for formula C_17_H_16_N_4_O_4_S [M+Na]^+^ 373.097, found [M+H]^+^ 373.100.
[2-(cyclohexylamino)-2-oxo-1-(4-phenyl-2-thienyl)ethyl] 5-amino-3-methyl-isoxazole-4-carboxylate (**PUB15**)
10% yield; m.p. 196–198 °C, beige solid. ^1^H NMR (600 MHz, DMSO-*d*_6_) δ (ppm): 1.02–1.31 (m, 5H), 1.45–1.82 (m, 6H), 2.24 (s, 3H, CH_3_ group of isoxazole ring), 3.49–3.60 (m, 1H), 6.30 (s, 1H), 7.28–7.33 (m, 1H), 7.40–7.45 (m, 2H), 7.65 (bs, 2H, NH_2_ group from isoxazole ring), 7.79–7.91 (m, 3H), 8.28 (d, *J* = 7.8 Hz, 1H). ^13^C NMR (151 MHz, DMSO-*d*_6_) δ (ppm): 12.34, 24.90, 25.57, 32.46, 32.54, 48.31, 70.69, 84.69, 122.46, 126.30, 127.78, 129.47, 135.26, 139.83, 141.00, 159.42, 161.67, 166.73, 127.47. ESI-MS: *m*/*z* calculated for formula C_23_H_25_N_3_O_4_S [M−H]^−^ 438.149, found [M−H]^−^ 438.161.
2-(cyclohexylamino)-1-(5-methylthiophen-2-yl)-2-oxoethyl 5-amino-3-methyl-1,2-oxazole-4-carboxylate (**PUB16**)
10% yield; m.p. 203–204 °C, beige solid. ^1^H NMR (600 MHz, DMSO-*d*_6_) δ (ppm): 1.35–1.02 (m, 5H), 1.81–1.49 (m, 5H), 2.21 (s, 3H), 2.42 (s, 3H), 3.44–3.55 (m, 1H), 6.18 (s, 1H), 6.70 (s, 1H), 6.98 (s, 1H), 7.82 (s, 2H, NH_2_ group from isoxazole ring), 8.18 (d, *J* = 7.8 Hz, 1H). ^13^C NMR (151 MHz, DMSO-*d*_6_) δ (ppm): 12.27, 15.43, 24.80, 25.58, 32.48, 32.56, 48.24, 70.72, 85.75, 125.31, 127.68, 136.19, 141.01, 159.43, 161.66, 166.87, 172.40. ESI-MS: *m*/*z* calculated for formula C_18_H_23_N_3_O_4_S [M−H]^−^ 376.134, found [M−H]^−^ 376.154.
2-(benzylamino)-1-(5-methylthiophen-2-yl)-2-oxoethyl 5-amino-3-methyl-1,2-oxazole-4-carboxylate (**PUB17**)
12% yield; m.p. 185–186 °C, light brown solid. ^1^H NMR (600 MHz, DMSO-*d*_6_) δ (ppm): 2.23 (s, 3H), 2.43 (s, 3H), 4.30 (s, 3H), 6.26 (s, 1H), 6.71 (s, 1H), 7.01 (s, 1H), 7.12–7.37 (m, 6H), 7.85 (s, 2H, NH_2_ group from isoxazole ring), 8.81 (t, *J* = 6.0 Hz, 1H). ^13^C NMR (151 MHz, DMSO-*d*_6_) δ (ppm): 12.38, 15.45, 42.49, 71.09, 84.73, 125.35, 127.29, 127.49, 127.97, 128.72, 135.77, 139.33, 141.19, 159.45, 161.80, 168.13, 172.49. ESI-MS: *m*/*z* calculated for formula C_19_H_19_N_3_O_4_S [M+H]^+^ 386.117, found [M+H]^+^ 386.117.
2-(benzylamino)-1-(1H-imidazol-4-yl)-2-oxoethyl 5-amino-3-methyl-1,2-oxazole-4-carboxylate (**PUB18**)
16% yield; m.p. 195–196 °C, beige solid. ^1^H NMR (600 MHz, DMSO-*d*_6_) δ (ppm): 2.19 (s, 3H), 4.41–4.22 (m, 2H), 6.08 (s, 1H), 7.18–7.32 (m, 5H), 7.68 (s, 1H), 7.86 (s, 2H, NH_2_ group from isoxazole ring), 8.62 (t, *J* = 6.4 Hz, 1H), 12.17 (s, 1H). ^13^C NMR (151 MHz, DMSO-*d*_6_) δ (ppm): 12.08, 42.42, 49.06, 85.06, 127.13, 127.42, 128.63, 139.67, 159.78, 161.97, 172.25. ESI-MS: *m*/*z* calculated for formula C_17_H_17_N_5_O_4_ [M+H]^+^ 356.135, found [M+H]^+^ 356.138.

### 2.3. Biological Assessments

**Figure 3:**
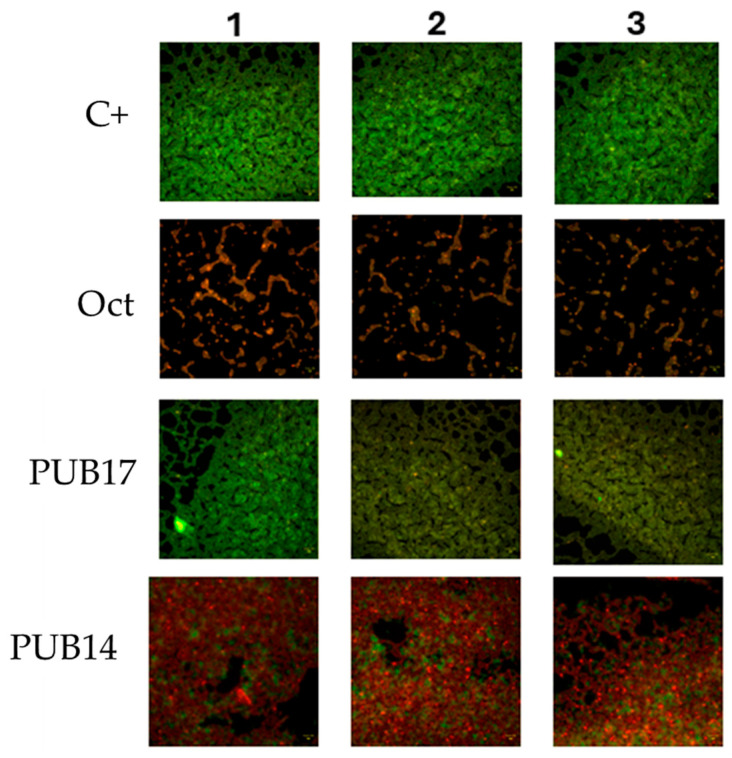
Visualization of *C. albicans* biofilm exposed to **PUB17** and **PUB14** compounds. C+ is a biofilm exposed to saline; “Oct” is a *C. albicans* biofilm exposed to octenidine dihydrochloride, a compound of known anti-biofilm activity. Performed in 3 repeats (1–3). Red shapes are dead *C. albicans* biofilm-forming cells; green shapes are live *C. albicans* biofilm-forming cells. Black shapes are areas devoid of live and dead cells. Live/dead Biofilm visualization kit; objective × 20; LumaScope 620 magnification × 20 (EtaLuma, San Diego, CA, USA).

In the first step, the minimum inhibitory concentration (MIC) of *S. aureus*, *P. aeruginosa* and *C. albicans*, as representatives of Gram-positive, Gram-negative bacteria and yeast-like fungus, respectively, were determined by a microdilution method to evaluate the antibacterial or/and antifungal activities of compounds **PUB11**–**PUB18**. The MIC values of the series of compounds are shown in Table 1. To our delight, we found that two compounds (**PUB14** and **PUB17**) exhibited antifungal properties, whereas the remaining compounds tested were inactive against bacteria and fungus. The formation of the *C. albicans* biofilm is one of the most important mechanisms of tolerance to drugs displayed by this microorganism. Compound **PUB14** showed strong anti-*Candida* properties and eradicated basically 55% of the biofilm formed by *C. albicans*. To establish our findings in a practically oriented context, we also tested the impact of octenidine dihydrochloride (a cationic antiseptic known of its antibiofilm properties) as the control drug against *C. albicans* ATCC 10,231 (Figure 3).

The quantitative data obtained by means of the microdilution method was confirmed by the visualization of live/dead biofilm-forming cells, showing the stronger effect of **PUB14** than **PUB17** towards *C. albicans* (Figure 3).

This selective type of effect, manifesting in antifungal, but not antibacterial efficacy, gave us idea of potential translational applicability compounds **PUB14** and **PUB17** in the treatment of vulvovaginal candidiasis. To develop this line of research, another set of microbial bacteria, namely *L. crispatus* and *L. gasseri*, constituting healthy vaginal microbiota were exposed to the set of **PUB14** and **PUB17** concentrations (Table 2).

Additionally, to test the intraspecies variability of yeast-like fungi on **PUB14** and **PUB17**, we have applied an additional 10 strains of *C. albicans*, isolated from various infections, and exposed their pre-formed biofilm on the aforementioned compounds. The average percentage reduction of candidal biofilm was 59.13 ± 10.73% and 35.06 ± 10.04% for **PUB14** and **PUB17**, respectively.

**Figure 4:**
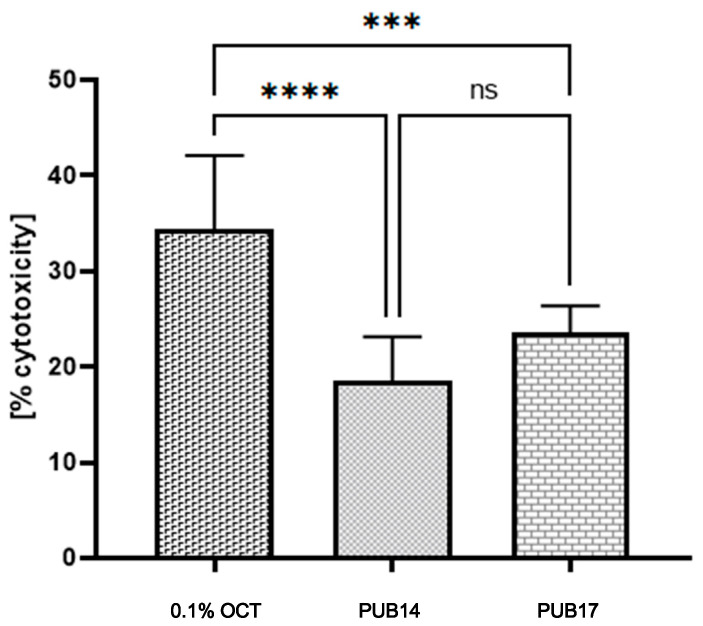
% cytotoxicity of **PUB14**, **PUB17** and 0.1% octenidine dihydrochloride towards HeLa cells. Asterisks indicate statistically significant differences between levels of cytotoxicity in analyzed compounds, ns—lack of statistical differences (*p* < 0.0001, ANOVA/Brown–Forsythe test for multiple comparisons).

Bearing in mind that potential clinical application of these compounds correlates with their exposure on eukaryotic cells in female patients, we performed the last series of investigation, i.e., we assessed the cytotoxicity of **PUB14** and **PUB17**, as well as octenidine dihydrochloride, towards HeLa cells in the clinically relevant exposure time of 15 min (Figure 4).

The obtained results indicated that both **PUB14** and **PUB17** compounds exhibited significantly lower cytotoxicity than 0.1% octenidine dihydrochloride (a concentration commonly used in commercial products for treating vaginal infections). Moreover, while the differences in cytotoxicity levels between **PUB14** and **PUB17** were noticeable, they were statistically insignificant, hovering around a favorable value of 20% (Figure 4).

## 3. Discussion

Five-membered rings consisting of one or more heteroatom (pyrazoles, imidazoles, thiazoles, etc.)-based analogs are the leading structure for the synthesis of its numerous derivatives, which have been shown to possess a wide range of pharmacological activities, and exhibited a very important role in development of many drugs [30,31,32]. They have been identified as excellent growth inhibitors of MRSA (methicillin-resistant *S. aureus*) [33]. In view of this, many structurally diversified heterocyclic hybrids were designed, synthesized and screened for their in vitro and in vivo efficacy to act against MRSA.

Our previous data show that isoxazole derivatives with various heterocyclic rings attached to their core display antimicrobial activities [17]. Our earlier studies revealed that the antimicrobial activity of two derivatives, 2-(cyclohexylamino)-1-(5-nitrothiophen-2-yl)-2-oxoethyl5-amino-3-methyl-1,2-oxazole-4- carboxylate (**PUB9**) and 2-(benzylamino)-1-(5-nitrothiophen-2-yl)-2-oxoethyl 5-amino-3-methyl-1,2oxazole-4-carboxylate (**PUB10**), was noticeably higher compared to the other compounds analyzed, especially **PUB9** with regard to *Staphylococcus*. The **PUB9** and **PUB10** derivatives were able to reduce more than 90% of biofilm-forming cells (*S. aureus*, *P. aeruginosa* and *C. albicans*) displaying at the same time no (**PUB9**) or moderate (**PUB10**) cytotoxicity in vitro. The analysis of the literature on the anti-biofilm activity of heterocyclic compounds evidenced few data and allowed us to identify the most frequently occurring heterocyclic and non-heterocyclic moieties in antifungal agents. Molecular hybridization is one of the most commonly used methods to develop new compounds, based on creating new effective molecules by using pharmacophore groups of two or more rings with known activity. The main benefit of employing an isoxazole ring into new structures is due to the fact that this moiety is a key pharmacophore for the antibacterial activity of many drugs such as sulfathiazole, sulfaphenazole or sulfamethoxazole. It was shown in the paper of Maillard [34] and others [18,26], that the presence of thiazole moiety enhanced the in vitro anti-*Candida* activity with an activity spectrum towards *Candida albicans* strains. The antifungal activity against clinically relevant *Candida* sp. strains was described for many small molecules bearing thiazole scaffolds [23,35]. In this study, all the compounds carry the common isoxazole ring and also the 1,3-thiazole, imidazole or thiophen moiety.

The main structural features identified in the family of 1,3-thiazole derivatives with antifungal activity by pharmacophore-based approaches include an aromatic nitrogen fragment with an accessible lone electron pair, functioning as a hydrogen bond acceptor, and a hydrophobic area represented by a phenyl ring [26]. Additionally, it was shown that the high lipophilicity of the thiazole derivatives was related to their high antifungal activity [18]. Because of the recently reported data regarding the importance of different heterocyclic moieties attached to the isoxazole ring in terms to improve the antimicrobial activity [11,17], we were encouraged to work toward the chemical development of a second series of new heterocyclic hybrids derived from 5-amino-3-methylisoxazole-4 carboxylic acid. Thus, we decided to incorporate other heterocyclic ring to obtain novel isoxazole-based derivatives to find the relationship between structure and antimicrobial activity in this class of compounds. Based on the literature review regarding the antifungal activity of different compounds, and considering the most common pharmacophores and the effect of lipophilicity of the thiazole derivatives, we decided to design and synthesize a series of model isoxazole-based heterocycles which may present antifungal properties towards *Candida albicans*. It resulted in the synthesis of the series of **PUB11**–**PUB18** derivatives, their chemical characterization and antimicrobial investigation. As shown in Table 1, Figure 3 and Table 2, the compounds **PUB14** and **PUB17** did not display activity against the tested bacterial species, while they did display this against tested 11 (in total) different strains of *C. albicans*. This selective antifungal activity of 2-(benzylamino)-2-oxo-1-(1,3-thiazol-2-yl)ethyl 5-amino-3-methyl-1,2-oxazole-4-carboxylate (**PUB14**) and 2-(benzylamino)-1-(5-methylthiophen-2-yl)-2-oxoethyl 5-amino-3-methyl-1,2-oxazole-4-carboxylate (**PUB17**) against *Candida albicans* is likely due to its interaction with fungal-specific components, such as ergosterol synthesis enzymes or cell wall synthesis pathways. Ergosterol is a crucial component of fungal cell membranes, lacking a direct counterpart in bacterial cells, thus representing an optimal target for antifungal medications. The observed activity may suggest that the compound either directly inhibits key enzymes involved in ergosterol biosynthesis, such as lanosterol 14α-demethylase, or interferes with upstream metabolic precursors. Further studies, including binding assays targeting enzymes such as lanosterol 14α-demethylase and β-1,3-glucan synthase, could provide insights into the mechanism of action of the compound, and genetic analyses of resistant fungal strains could reveal adaptive mutations in targeted pathways, while transcriptomic studies that might identify changes in fungal gene expression after treatment are required to elucidate the precise mechanism of action [36,37].

The compounds’ unique structural features, including the oxazole and thiophene/thiazole rings, may facilitate these interactions, leading to fungal cell death while sparing bacterial cells. Additionally, the calculated lipophilicity of **PUB14** and **PUB17** compounds (logP values are 2.236 and 3.150, respectively) meet lipophilicity criteria for their antifungal activity according to the data presented by Biernasiuk et al. [18]. Further studies, including binding assays and genetic analyses, are required to elucidate the precise mechanism of action. The observed selective antifungal activity of the synthesized compounds against *Candida albicans*, coupled with its lack of antibacterial effects and minimal cytotoxicity toward HeLa cells (Figure 4), suggests a targeted mechanism of action. This specificity may be attributed to the compound’s interaction with cellular components or pathways unique to fungi and mammalian cells, which are absent in bacteria. One plausible explanation is the inhibition of ergosterol biosynthesis, a critical component of fungal cell membranes. While bacteria do not synthesize sterols, mammalian cells produce cholesterol, a structural analog of ergosterol. The compounds’ weak cytotoxicity toward HeLa cells could result from partial inhibition of cholesterol biosynthesis or interference with sterol-dependent cellular processes. Additionally, the compounds may disrupt mitochondrial function, as mitochondria in both fungi and mammalian cells share similarities in their electron transport chains and membrane composition. Such disruption could lead to apoptosis or cell death in these cell types, while bacterial cells, lacking mitochondria, remain unaffected [38,39,40].

The results obtained in this study provide valuable insights and substantially contribute to the current understanding within this field. These findings align with previous observations and literature reports, offering a coherent explanation of the observed phenomena. A notable strength of this work lies in its elucidation of the intricate relationship between the chemical structure and the biological activity of the analyzed derivatives. This highlights the potential for further exploration in this area, particularly through the design of novel derivatives using advanced chemical synthesis and computational chemistry approaches. Such efforts could pave the way for additional discoveries of significant scientific merit.

Considering the need for searching new antifungal agents and the discovery of an antifungal potential drug that does not inhibit lactobacilli growth could be a potential strategy to prevent and combat vulvovaginal candidiasis.

## 4. Materials and Methods

### 4.1. Chemistry

Commercially available reagents, i.e., triethyl orthoacetate, cyanoacetate, hydrazine monohydrate, aldehydes, isocyanides, were purchased from TCI (Tokyo, Japan) and Sigma-Aldrich (Merck Group, Darmstadt, Germany), and were used without further purification. Thin layer chromatography (TLC) was used to assess the progress and completion of reactions, and was carried out using Alugram SIL G/UV254/365 nm plates (Macherey-Nagel, Düren, Germany) and the developing system ethyl chloroform/methanol = 9/1 (*v*/*v*) and visualized by ultraviolet (UV) light at (254 nm and/or 365 mm) (UV A. KRÜSS Optronic GmbH, Hamburg, Germany). Melting points were determined by uniMELT 2 apparatus (LLG, Meckenheim, Germany) and were uncorrected.

^1^H and ^13^C NMR were recorded at Bruker NMR AVANCE III™ 600 MHz spectrometer. ^1^H and ^13^C NMR chemical shifts were referenced to the solvent signal, i.e., for ^1^H: δ (quintet of residual DMSO-*d*_6_) = 2.50 ppm, and for ^13^C: δ (non-decoupled septet of DMSO-*d*_6_) = 39.52 ppm. NMR spectral analyses were performed using MestReNova (Mnova version 14.2) software (Mestrelab Research S.L., Santiago de Compostela, Spain).

The following abbreviations are used for the multiplicities: s: singlet, d: doublet, t: triplet, q: quartet, m: multiplet, br s: broad singlet for proton spectra. Coupling constants (*J*) are reported in Hertz (Hz).

The ESI–MS spectra were recorded with the compactTM Bruker Daltonics Electrospray Ionization Quadrupole Time-of-Flight (ESI-Q-TOF) apparatus (Bruker Daltonics GmbH, Bremen, Germany). Analysis was performed in the positive and negative ion mode between 100 and 3000 *m*/*z*. MS parameters were the following: the nebulizing gas was nitrogen, the nebulizing gas flow was 3.0 L/min, the drying gas flow was 10 L/min, the heating gas flow was 10 L/min, interface temperature was 300 °C, desolvation line temperature was 400 °C, detector voltage was 2.02 kV, interface voltage was 4.0 kV, collision gas was argon. Bruker Compass Data Analysis 4.2 software (Bruker Daltonics GmbH, Bremen, Germany) was used for ESI–MS spectral analysis and calculation of the theoretical monoisotopic mass of detected ions. Flash column chromatography was performed using Büchi Pure Chromatography C-900 system with pure solvents. The following programs were used to calculate LogP: ChemSketch (ACDLabs software version 12.01), and ADMETlab software [41].

### 4.2. Biological Assessments

The provided compounds were dissolved in 1% of dimethyl sulfoxide (ChemPur, Piekary Śląskie, Poland) with the highest concentration of 10 µg/mL. The following strains and eukaryotic cell line from ATTC (Manassas, VA, USA) were used: *S. aureus* 29,213, *P. aeruginosa* 115,442, *C. albicans* 10,231, *L. crispatus* 33,197, *L. gasseri* 19,992 and HeLa (CCL-2).

#### 4.2.1. Determination of Minimum Inhibitory Concentration (MIC), Minimal Biocidal Concentration, Minimum Biofilm Eradication Concentration (MBEC) or % Reduction of Biofilms

The MIC of the analyzed compounds against tested microorganisms was determined using a standard resazurin-based microdilution method in microplate model [11]. A standardized inoculum of tested microorganisms (approximately 1 × 10^5^ CFU/mL) from an initial concentration of 0.5 McFarland (bacterial suspension) or 1 McFarland (fungal suspension) according to EUCAST was prepared in tryptic soy broth or, in case of *Lactobacilli*, in Man, Rogosa, and Sharpe broth (Biomaxima, Warsaw, Poland). In a sterile 96-well microtiter plate (BioStar, Munchen, Germany), 100 µL of the inoculum was added to each well containing 100 µL of serially diluted compounds. Control wells included a medium with inoculum (positive control) and a medium without inoculum (negative control). The plates were incubated at 37 °C for 24 h. Following incubation, 20 µL of 0.01% resazurin solution was added to each well, and the plates were further incubated for 2 h. The reduction of resazurin to resorufin by metabolically active cells was assessed by measuring the absorbance at 570 nm and 600 nm, using a microplate reader. The MIC was defined as the lowest concentration of the compound that resulted in inhibiting microbial growth. In case of MBC, the same procedure as in the case of MIC assessment was performed, with the difference that, after exposure on analyzed compounds, the whole content of wells was spotted on the appropriate agar plates—MRS for *lactobacilli*, and TSA for remaining microbiological species. In case of MBEC, biofilms of tested microorganisms were established by adding 100 µL of the standardized inoculum to each well of a 96-well plate and incubating them at 37 °C for 24 h. Non-adherent cells were removed by gently washing the wells once with 200 µL of saline. Serial dilutions of the compounds were prepared in respective broths, and 100 µL of each dilution was added to the respective wells containing the pre-established biofilms. The plates were incubated at 37 °C for 24 h. Following treatment, wells were washed once with saline to remove residual compounds. Then, 100 µL of 0.01% resazurin solution was added to each well, and the plates were incubated at 37 °C for 2 h. The metabolic activity of the biofilms was assessed by measuring absorbance at 570 nm and 600 nm. The MBEC was defined as the lowest concentration of the compound that resulted in a significant decrease in absorbance at 570 nm, indicating the eradication of the biofilm. All experiments were performed in triplicate. As no MIC**/**MBEC values (100% of eradication) were detected, absorbance readings were used to calculate the percentage reduction of resazurin, providing a semiquantitative measure of cell viability. The percentage reduction was calculated using the formula: [%] reduction of biofilm = absorbance value of control (non-treated biofilm) sample − absorbance value of treated sample**/**absorbance value of control (non-treated biofilm) sample × 100.

#### 4.2.2. Visualization of the Impact of Analyzed Compounds on Candida Albicans Biofilm Using Fluorescence Microscopy

The biofilm cultivation and treatment with formulations were conducted as outlined in Section concerning MBEC assessment [42]. According to the manufacturer’s instructions, each sample was stained with 500 μL of the FilmTracer™ LIVE/DEAD™ Biofilm Viability Kit (Thermo Fisher Scientific, Waltham, MA, USA). The specimens were incubated in the dark for 20 min, after which the staining solution was gently removed. The samples were then rinsed once with sterile filtered water. These prepared specimens were subsequently analyzed using an LS620 widefield fluorescence microscope (Etaluma, San Diego, CA, USA) equipped with a 20× objective lens.

#### 4.2.3. Assessment of In Vitro Cytotoxicity Towards HeLa Cell Line [43]

HeLa cells were cultured in Dulbecco’s Modified Eagle Medium (DMEM) (Radnor, PA, USA), supplemented with 10% fetal bovine serum (FBS) and maintained at 37 °C in an atmosphere containing 5% CO_2_. To assess the cytotoxicity of analyzed compounds, the neutral red uptake (NRU) assay was employed following established protocols. Cells were seeded in 96-well plates at a density of 1 × 10^4^ cells per well and incubated for 24 h to allow adherence. Subsequently, the culture medium was replaced with a fresh medium containing 10 µg/mL of **PUB14** or **PUB17** or 0.1% octenidine. Cells were exposed to the compound for 15 min at 37 °C. Following exposure, the treatment medium was removed, and cells were washed twice with phosphate-buffered saline (PBS). Neutral red solution (50 µg/mL in culture medium) was then added to each well, and plates were incubated for an additional 2 h. After incubation, cells were washed with PBS and fixed using a solution of 1% calcium chloride in 0.5% formaldehyde. The incorporated dye was extracted with a solution of 50% ethanol, 49% distilled water, and 1% glacial acetic acid (ChemPur, Piekary Śląskie, Poland). Absorbance was measured at 540 nm using a microplate reader. Cell viability was expressed as a percentage relative to untreated control cells. All experiments were performed in 6 repeats.

#### 4.2.4. Statistical Analysis

Statistical calculations were conducted with GraphPad Prism 10 (GraphPad Software, San Diego, CA, USA). The normality of distribution was checked with a Shapiro–Wilk test, then the ANOVA test with Brown–Forsythe test for multiple comparisons was performed.

## 5. Conclusions

The limited number of approved drugs for the treatment of *Candida albicans*-related infections and the rapid emergence of resistance to the existing antifungals have led to the search for novel antifungal agents. Both the literature analysis and the research results we have obtained so far regarding the antimicrobial properties of isoxazole derivatives encourage us to look for potential biologically active agents in this group of compounds. The distinct attributes of compounds **PUB14** and **PUB17**, including their anti-Candida properties, minimal cytotoxicity towards HeLa cells, and non-inhibition of lactobacilli growth, warrant further investigation as potent antifungal drug candidates.

## Figures and Tables

**Table 1:** The minimal inhibitory concentration [µg/mL] (MIC), the minimal biocidal concentration (MBC) and the minimal biofilm eradication concentration (MBEC) of tested compounds towards *S. aureus* 29,213, *P. aeruginosa* 115,442 and *C. albicans* 10,231, tested in the microplate model. The bold values indicate the percentage reduction of *C. albicans* exposed to the highest possible concentration of compound dissolved in 1% DMSO (which is a non-toxic concentration for the analyzed microorganisms).

	*S.aureus* ATCC 29,213			*P. aeruginosa* ATCC 115,442			*C. albicans* ATCC 10,231		
Compound	MIC [µg/mL]	MBC [µg/mL]	MBEC [% reduction]	MIC [µg/mL]	MBC [µg/mL]	MBEC [% Reduction]	MIC [µg/mL]	MBC [µg/mL]	MBEC [% Reduction]
**PUB11**	>10	>10	0%	>10	>10	0%	>10	>10	0%
**PUB12**	>10	>10	0%	>10	>10	0%	>10	>10	0%
**PUB13**	>10	>10	0%	>10	>10	0%	>10	>10	0%
**PUB14**	>10	>10	0%	>10	>10	0%	>10	>10	**54.9%**
**PUB15**	>10	>10	0%	>10	>10	0%	>10	>10	0%
**PUB16**	>10	>10	0%	>10	>10	0%	>10	>10	0%
**PUB17**	>10	>10	0%	>10	>10	0%	>10	>10	**24.6%**
**PUB18**	>10	>10	0%	>10	>10	0%	>10	>10	0%

**Table 2:** The minimal inhibitory concentration [µg/mL] (MIC), the minimal bactericidal concentration (MBC) and the minimal biofilm eradication concentration (MBEC) of compounds **PUB14** and **PUB17**, towards *L. crispatus* 33,197 and *L.gasseri* 19,992 tested in microplate model.

	*L. crispatus* 33,197			*L. gasseri* 19,992		
Compound	MIC [µg/mL]	MBC [µg/mL]	MBEC [% Reduction]	MIC [µg/mL]	MBC [µg/mL]	MBEC [% Reduction]
**PUB14**	>10	>10	0%	>10	>10	0%
**PUB17**	>10	>10	0%	>10	>10	0%

## Data Availability

All necessary data are presented in the manuscript and the raw data can be provided from the authors upon reasonable request.

## Supplementary Materials

The supporting information can be downloaded at: https://www.mdpi.com/article/10.3390/ijms252413618/s1.
