# Supplementary material for: Novel Isoxazole-Based Antifungal Drug Candidates
Source: Int J Mol Sci. 2024 Dec 19;25(24):13618. doi: 10.3390/ijms252413618 (PMC11728180; doi:10.3390/ijms252413618)
Supplement: Supplementary file 1 [file ijms-25-13618-s001.zip › ijms-3352380-supplementary.pdf]

SUPPLEMENTARY MATERIAL

Novel isoxazole-based antifungal drug candidates

Urszula Bąchor[1]*, Malwina Brożyna[2], Adam Junka[2], Mateusz Ramires Chmielarz[3], Damian Gorczyca[4], and Marcin Mączyński[1]*

[1] Department of Organic Chemistry and Drug Technology, Faculty of Pharmacy, Wroclaw Medical University, 50-556 Wroclaw, Poland
[2] Unique Application Model Laboratory, Department of Pharmaceutical Microbiology and Parasitology, Faculty of Pharmacy, Wroclaw Medical University, 50-556 Wroclaw, Poland
[3] Department of Clinical Microbiology, Faculty of Medicine, Wroclaw Medical University, Chalubinskiego 4, 50-368 Wroclaw, Poland
[4] Faculty of Medicine, Lazarski University, 02-662 Warszawa, Poland; damian.gorczyca@lazarski.pl
* Correspondence: urszula.bachor@umw.edu.pl (UB); marcin.maczynski@umw.edu.pl (MM)

*Corresponding authors: urszula.bachor@umw.edu.pl (UB); marcin.maczynski@umw.edu.pl (MM)

Citation: To be added by editorial staff during production.

Academic Editor: Firstname Lastname

Received: date
Revised: date
Accepted: date
Published: date

Copyright: © 2024 by the authors. Submitted for possible open access publication under the terms and conditions of the Creative Commons Attribution (CC BY) license (https://creativecommons.org/licenses/by/4.0/).

TABLE OF CONTENTS

1. CHEMISTRY

1.1 ^{1}H and ^{13}C NMR spectra of compound PUB11 3

1.2 ^{1}H and ^{13}C NMR spectra of compound PUB12 6

1.3 ^{1}H and ^{13}C NMR spectra of compound PUB13 9

1.4 ^{1}H and ^{13}C NMR spectra of compound PUB14 11

1.5 ^{1}H and ^{13}C NMR spectra of compound PUB15 13

1.6 ^{1}H and ^{13}C NMR spectra of compound PUB16 15

1.7 ^{1}H and ^{13}C NMR spectra of compound PUB17 17

1.8 ^{1}H and ^{13}C NMR spectra of compound PUB18 19

2. PHYSICOCHEMICAL PROPERTIES, PHARMACOKINETICS AND ADME ACTIVITY

1. CHEMISTRY

1.1 Analysis of ^{1}H NMR, ^{13}C NMR and ESI-MS spectra of compound PUB11

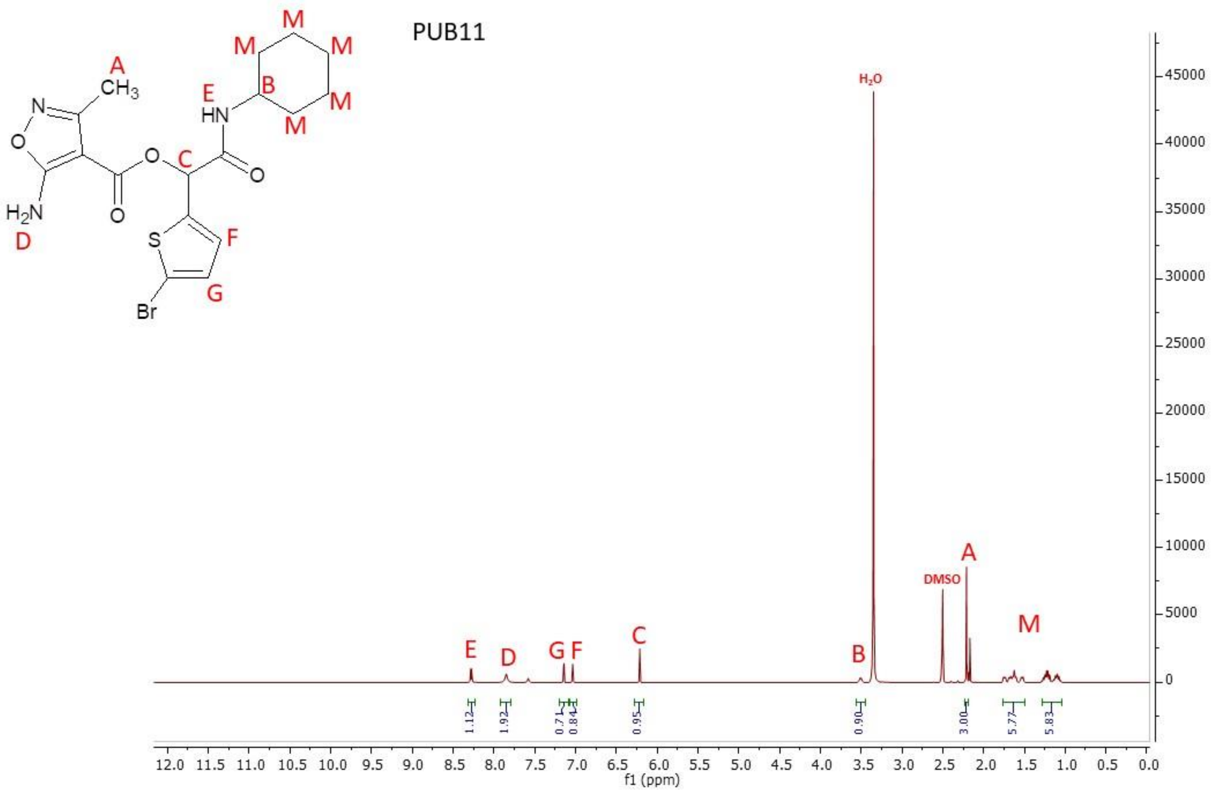

Figure S1. ^{1}H NMR spectrum of compound PUB11 in DMSO-d_6.

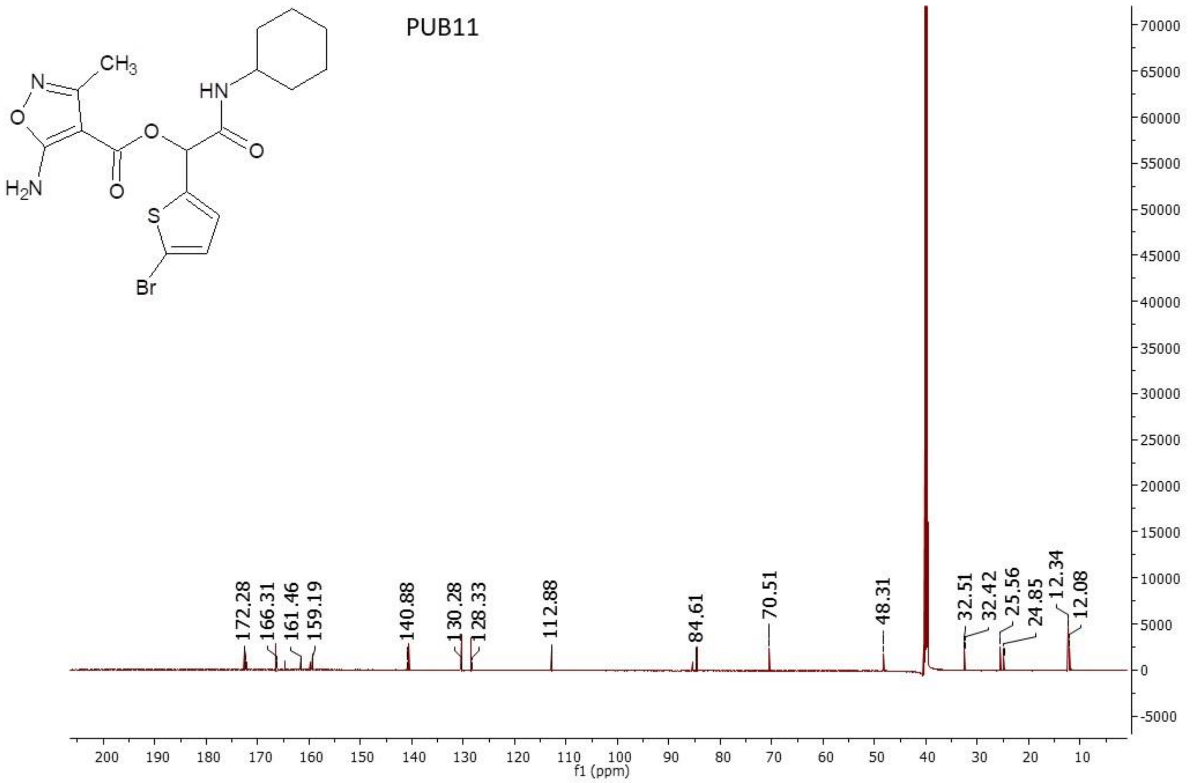

Figure S2. ^{13}C NMR spectrum of compound PUB11 in DMSO-d_6.

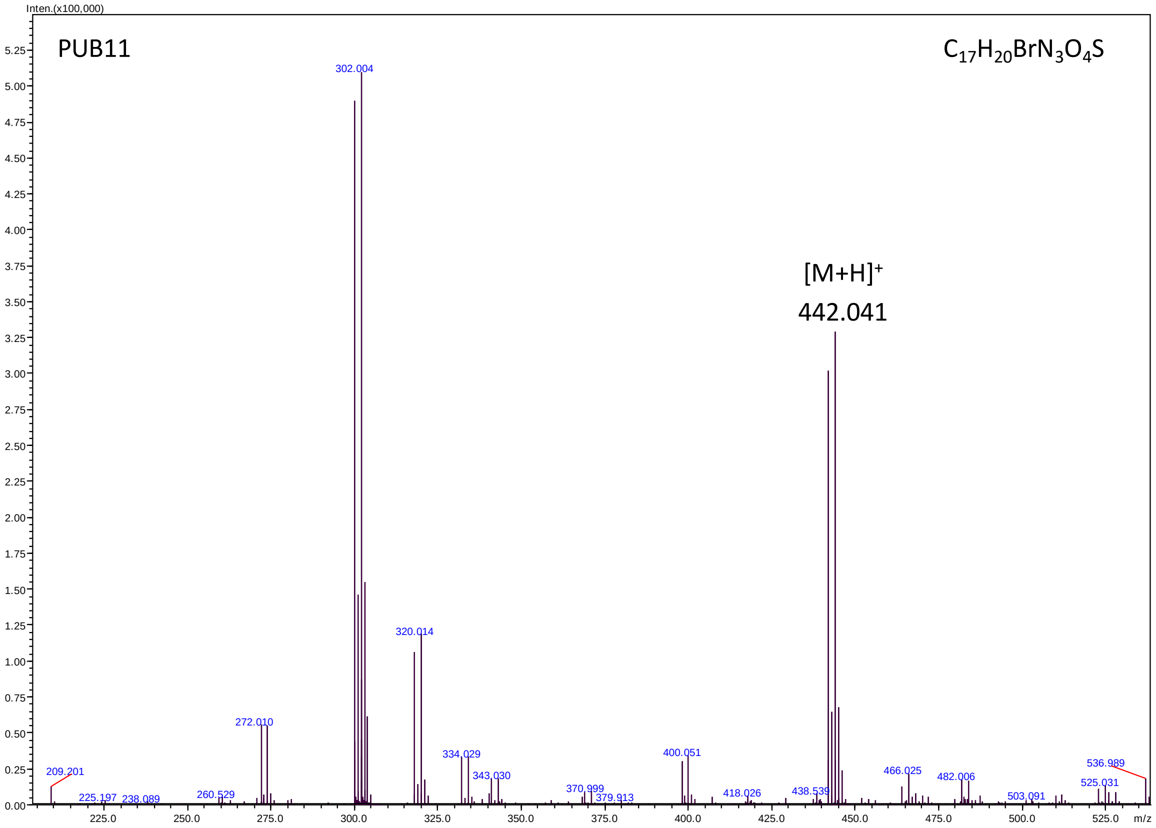

Figure S3. ESI-MS spectrum of compound PUB11.

1.2 Analysis of ^{1}H NMR, ^{13}C NMR and ESI-MS ^{1}H and ^{13}C NMR spectra of compound PUB12

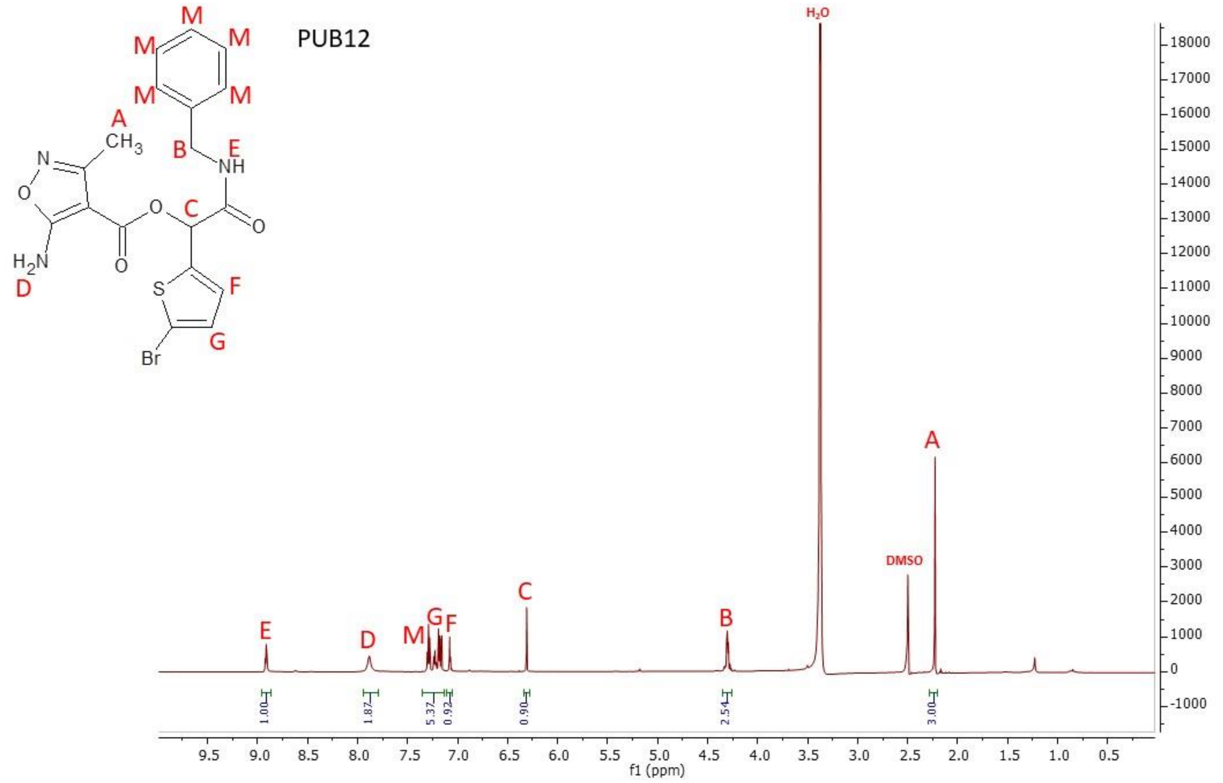

Figure S4. ^{1}H NMR spectrum of compound PUB12 in DMSO-d_6.

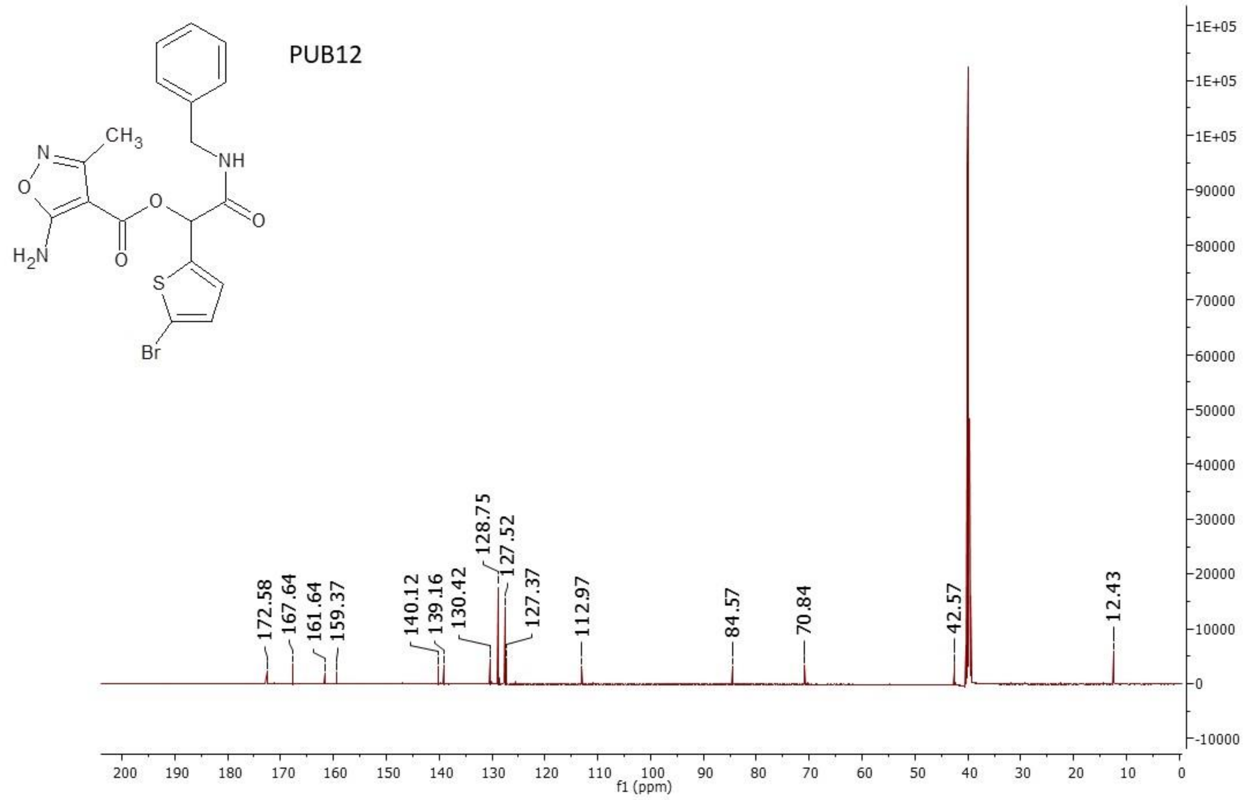

Figure S5. ^{13}C NMR spectrum of compound PUB12 in DMSO-d_6.

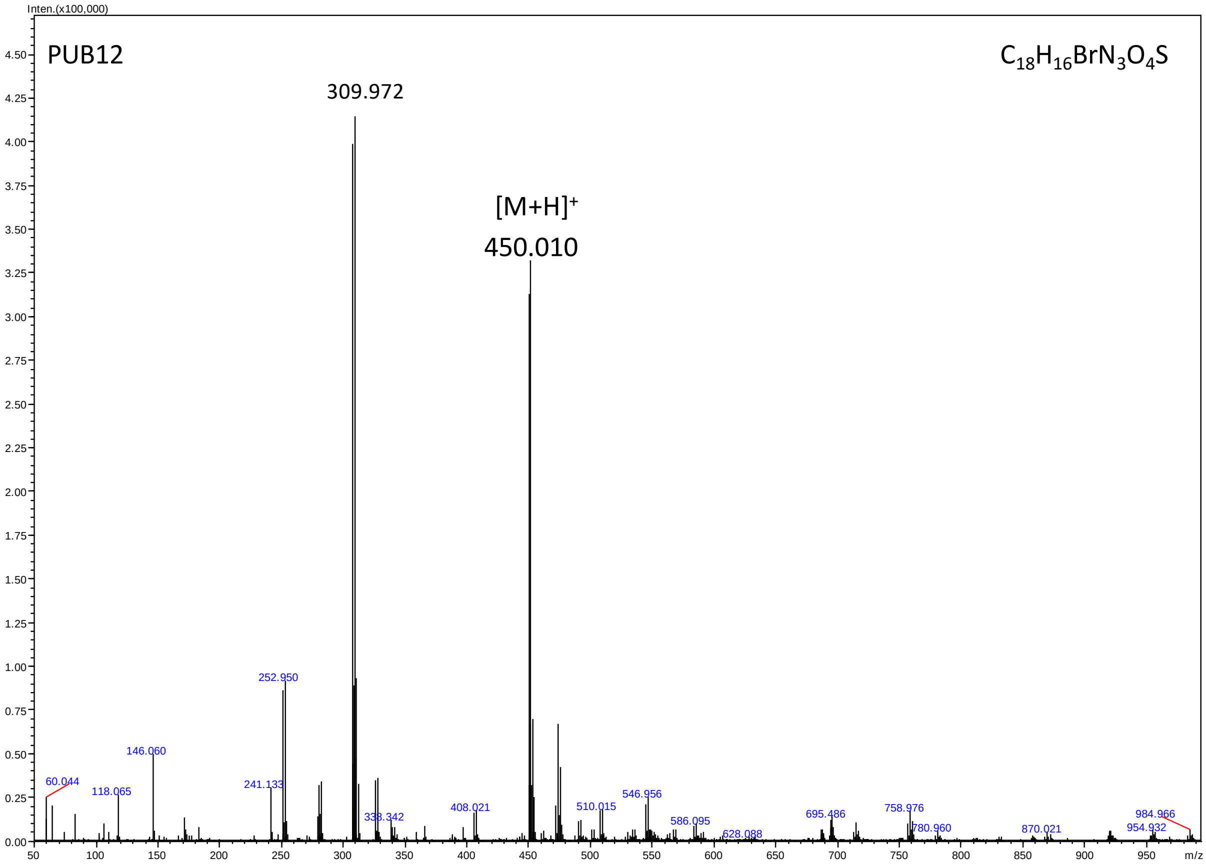

Figure S6. ESI-MS spectrum of compound PUB12.

1.3 Analysis of ^{1}H NMR, ^{13}C NMR and ESI-MS ^{1}H and ^{13}C NMR spectra of compound PUB13

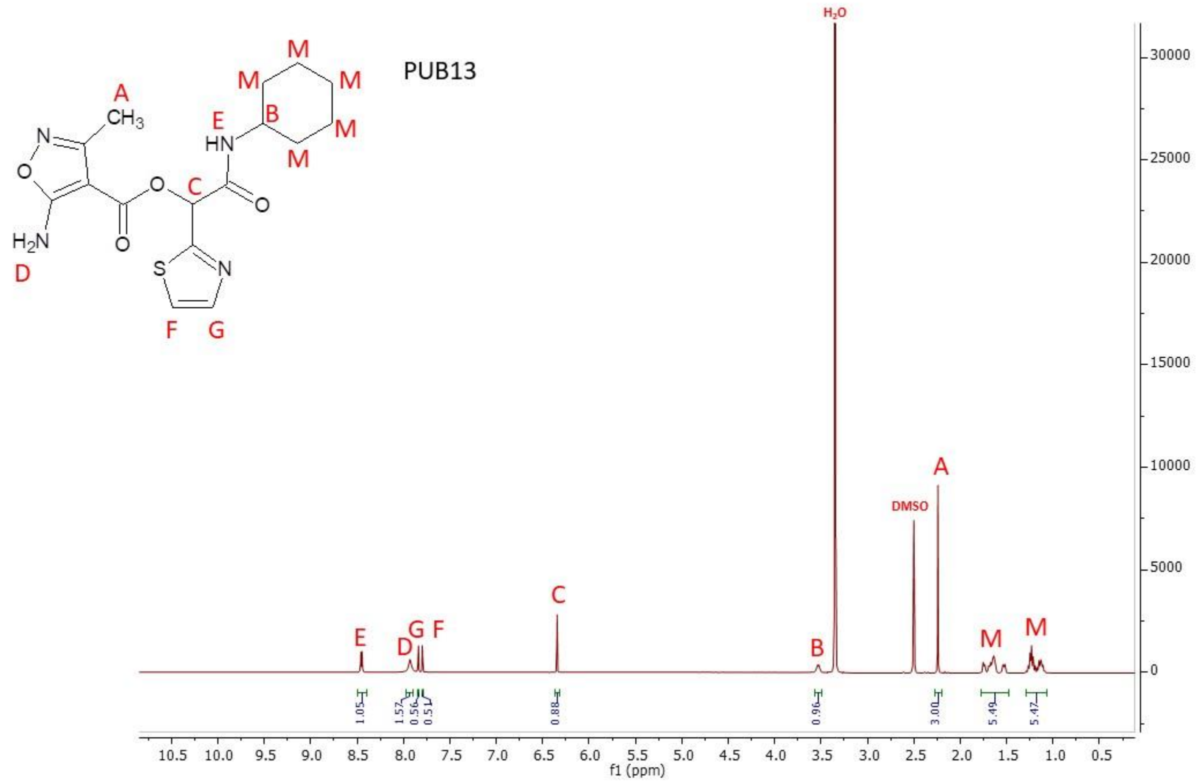

Figure S7. ^{1}H NMR spectrum of compound PUB13 in DMSO-d$_6$.

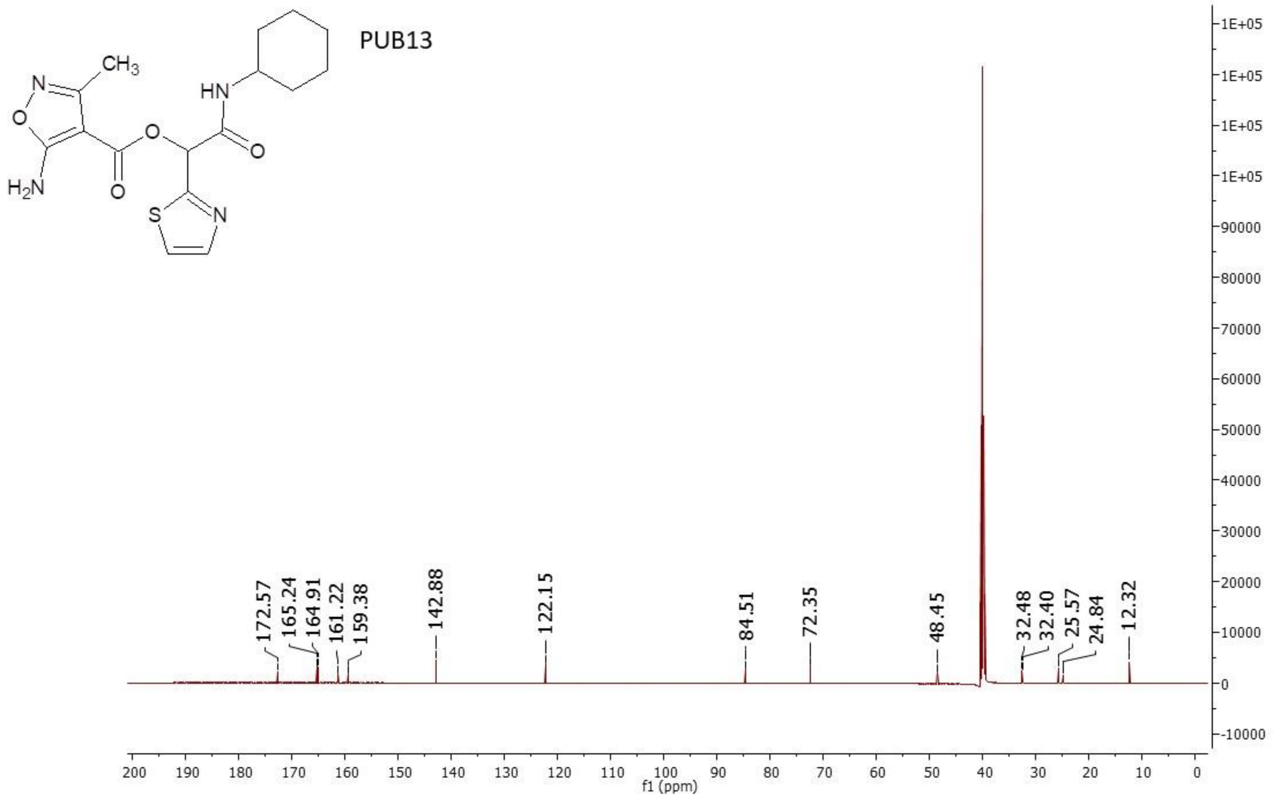

Figure S8. ^{13}C NMR spectrum of compound PUB13 in DMSO-d_6.

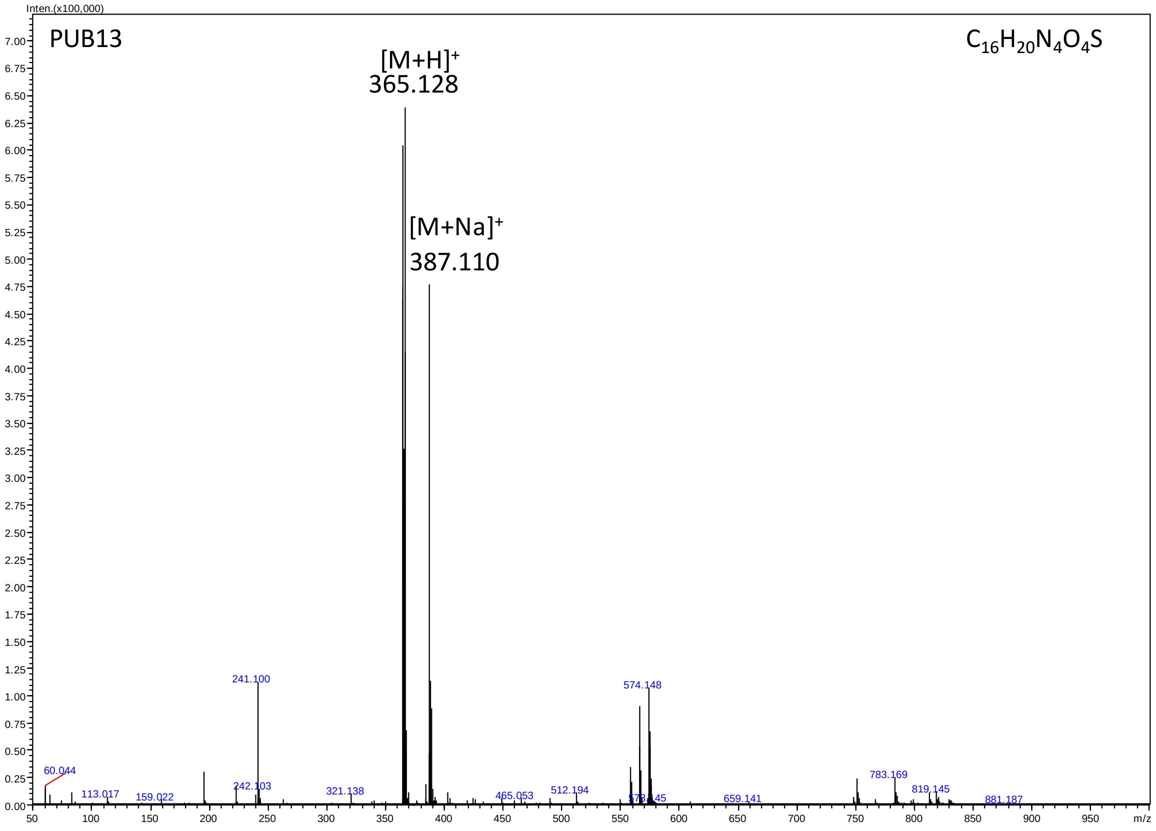

Figure S9. ESI-MS spectrum of compound PUB13.

1.4 Analysis of ^{1}H NMR, ^{13}C NMR and ESI-MS ^{1}H and ^{13}C NMR spectra of compound PUB14

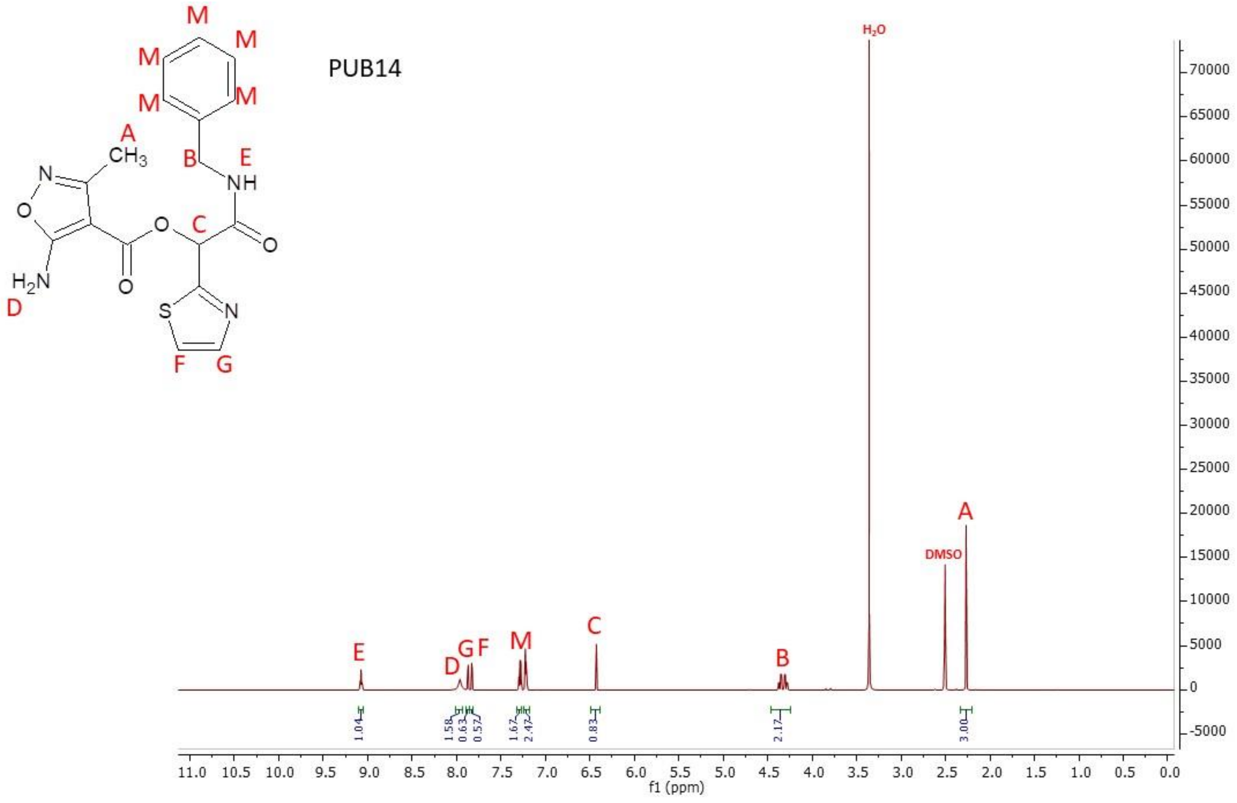

Figure S10. ^{1}H NMR spectrum of compound PUB14 in DMSO-d_6.

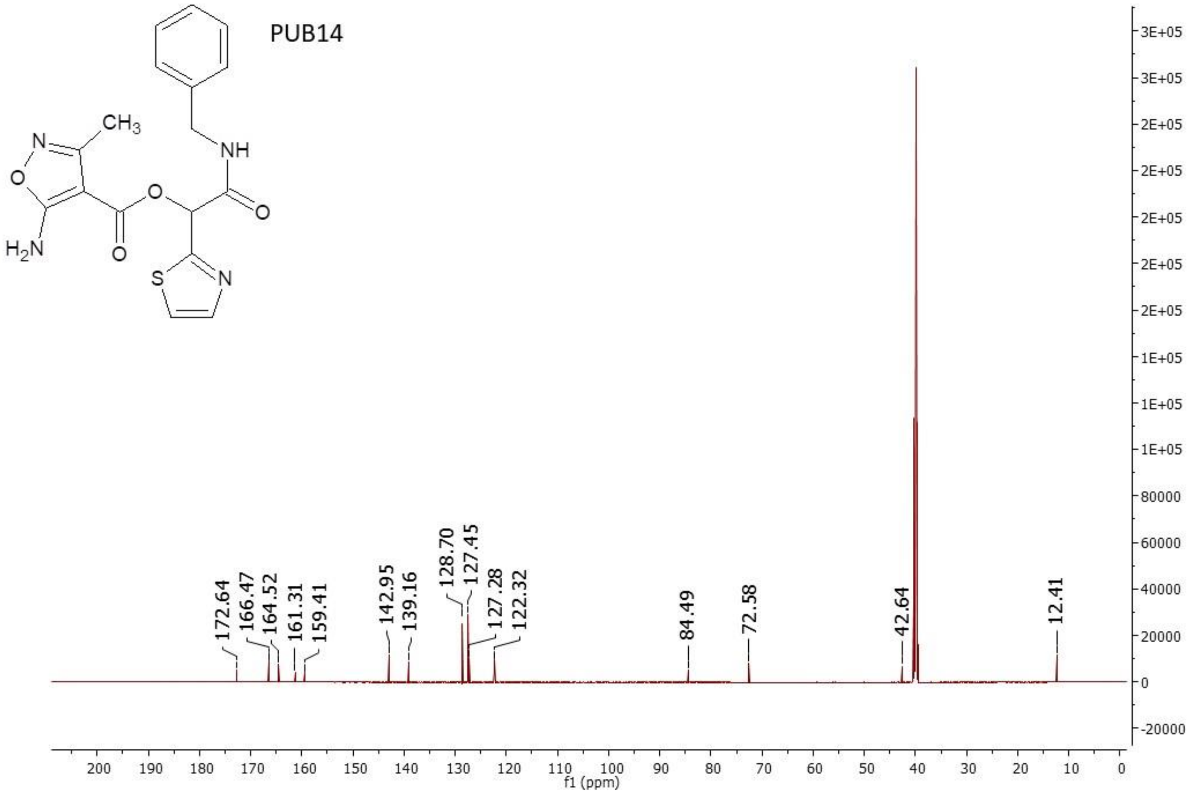

Figure S11. ^{13}C NMR spectrum of compound PUB14 in DMSO-d_6.

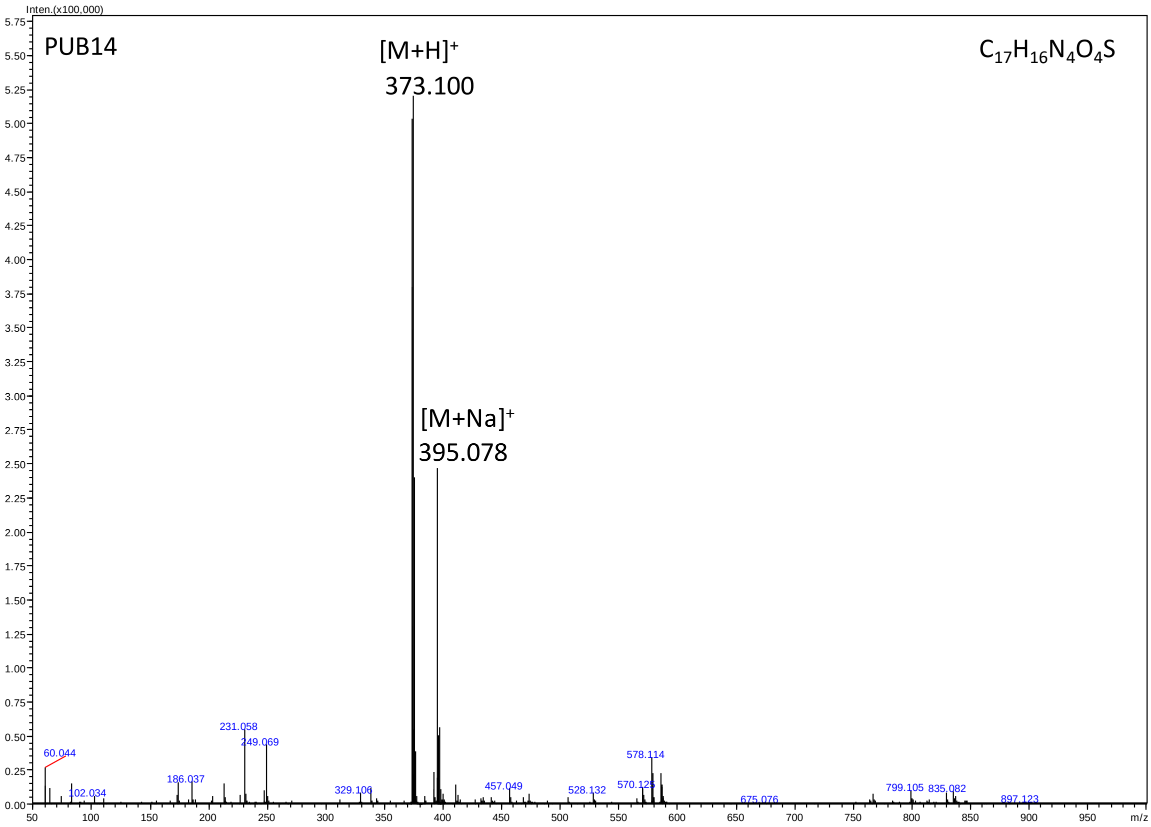

Figure S12. ESI-MS spectrum of compound PUB14.

1.5 Analysis of ^{1}H NMR, ^{13}C NMR and ESI-MS ^{1}H and ^{13}C NMR spectra of compound PUB15

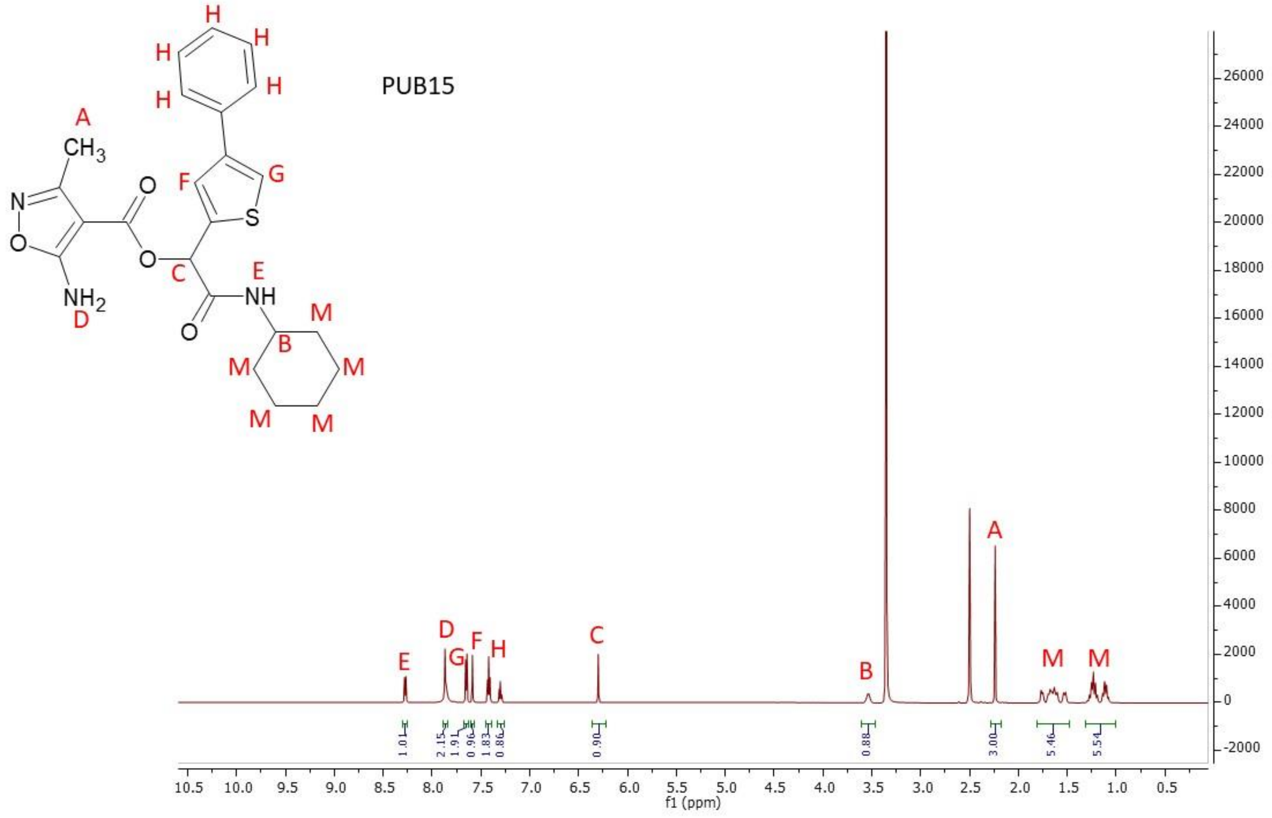

Figure S13. ^{1}H NMR spectrum of compound PUB15 in DMSO-d_6.

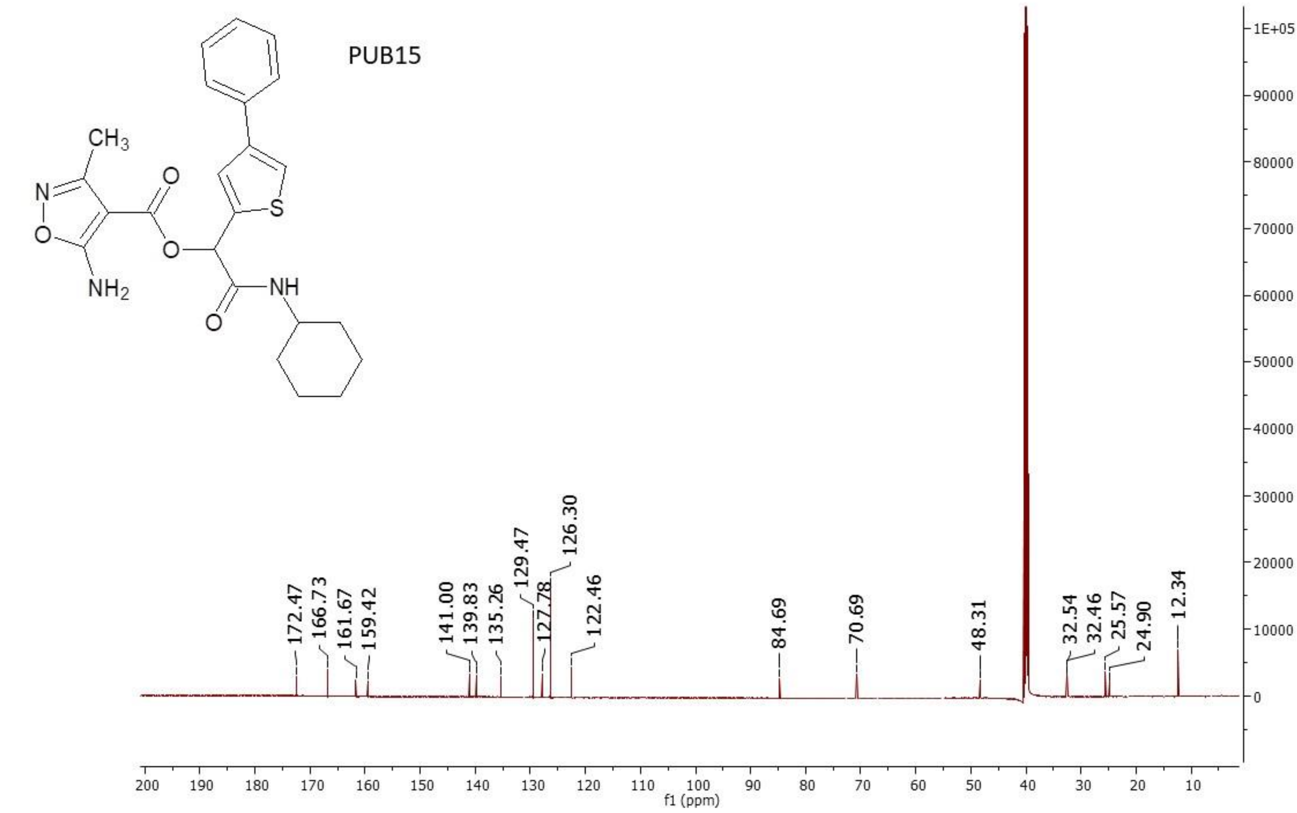

Figure S14. ^{13}C NMR spectrum of compound PUB15 in DMSO-d_6.

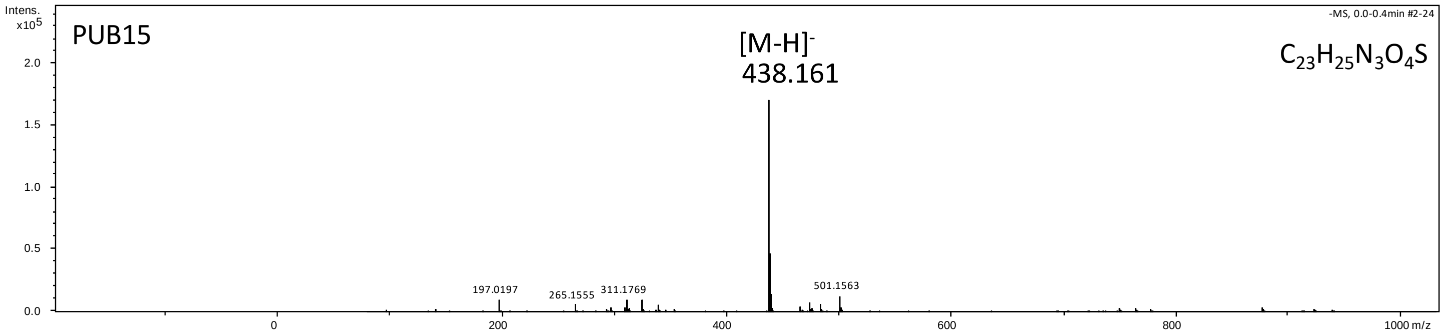

Figure S15. ESI-MS spectrum of compound PUB15.

1.6 Analysis of ^{1}H NMR, ^{13}C NMR and ESI-MS ^{1}H and ^{13}C NMR spectra of compound PUB16

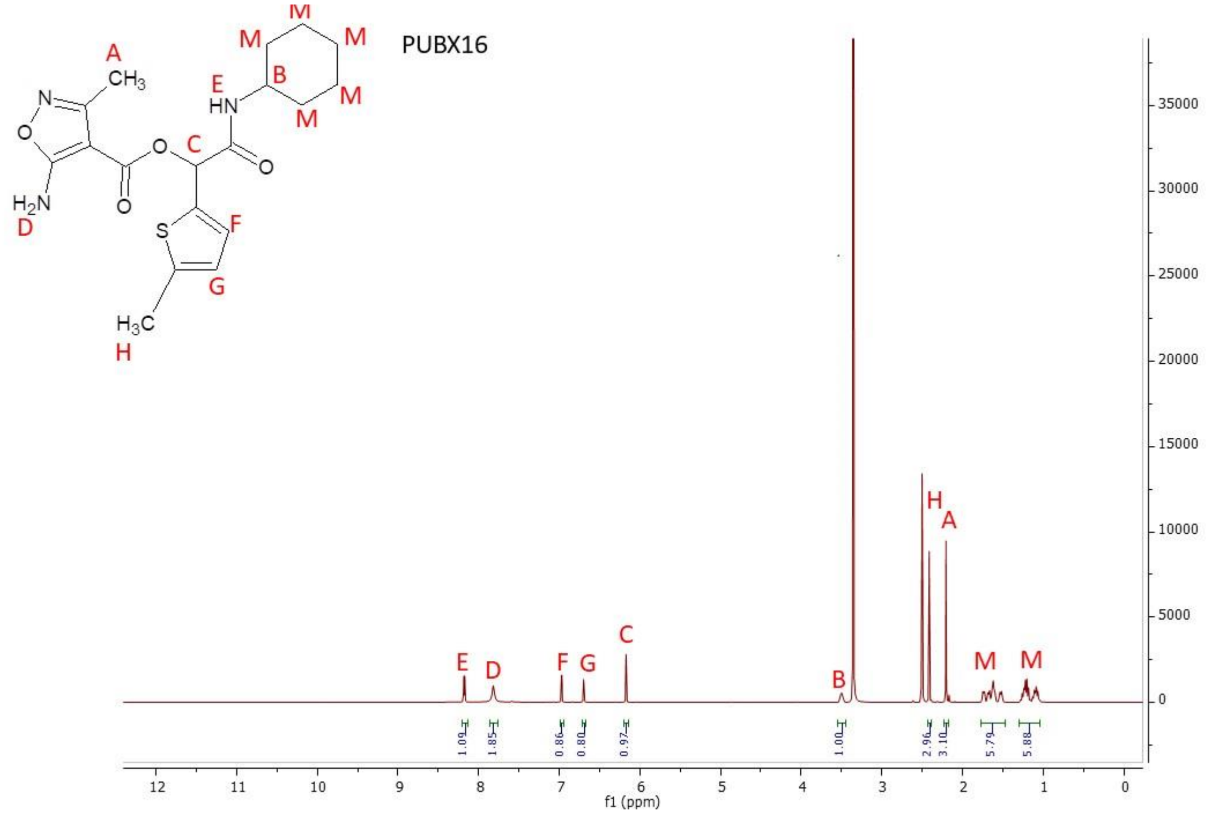

Figure S16. ^{1}H NMR spectrum of compound PUB16 in DMSO-d_6.

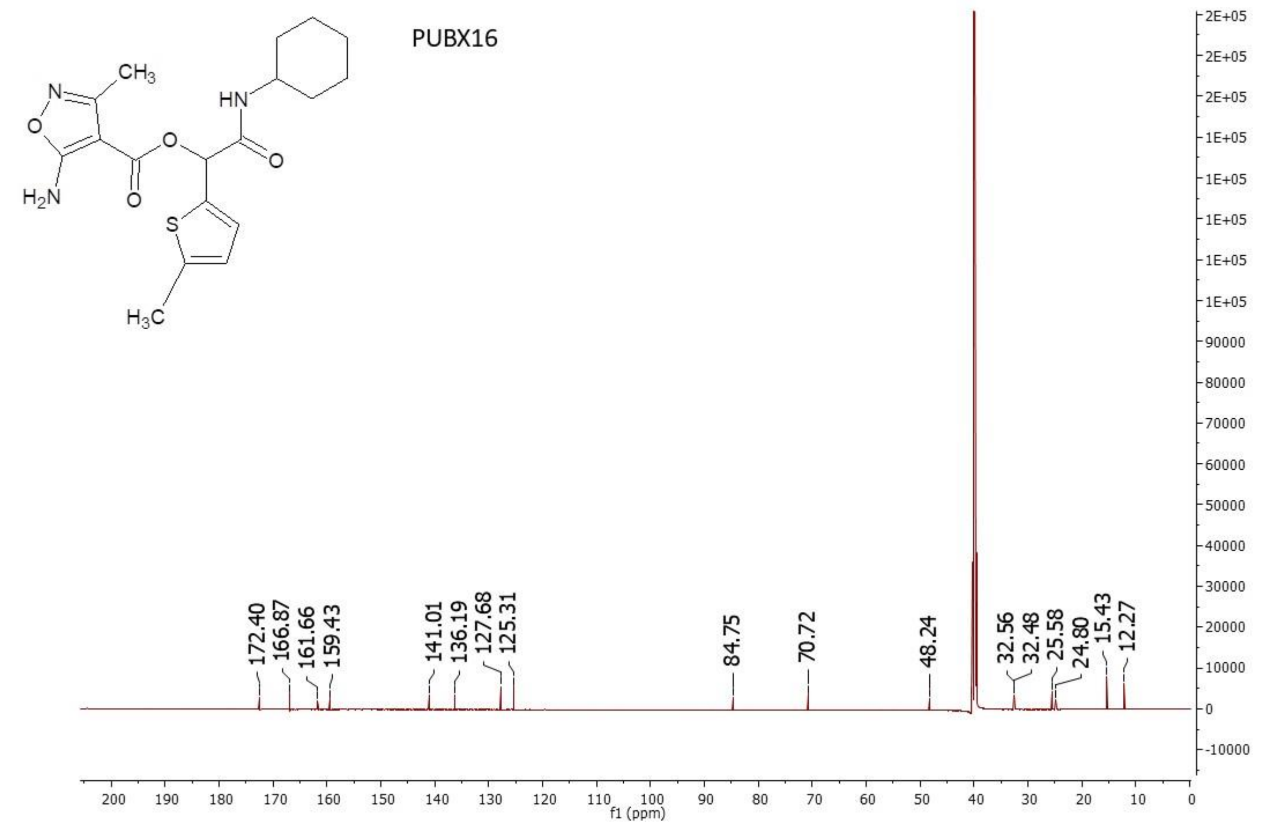

Figure S17. ^{13}C NMR spectrum of compound PUB16 in DMSO-d_6.

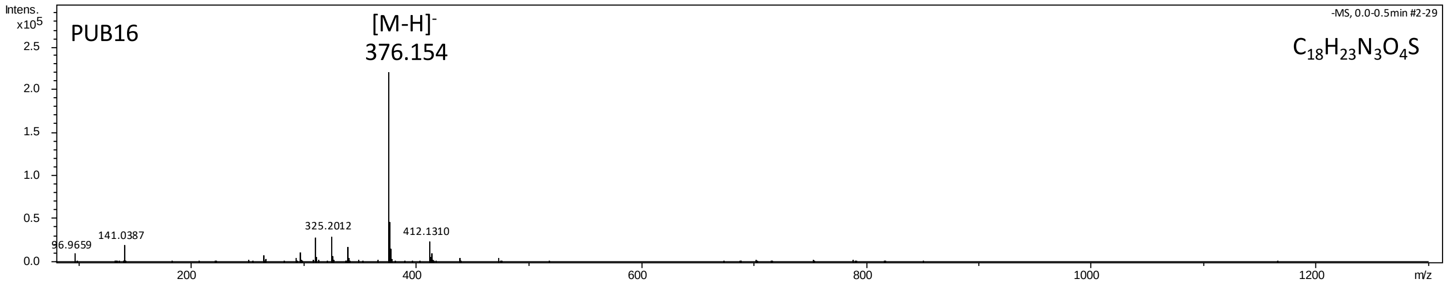

Figure S18. ESI-MS spectrum of compound PUB16.

1.7 Analysis of ^{1}H NMR, ^{13}C NMR and ESI-MS ^{1}H and ^{13}C NMR spectra of compound PUB17

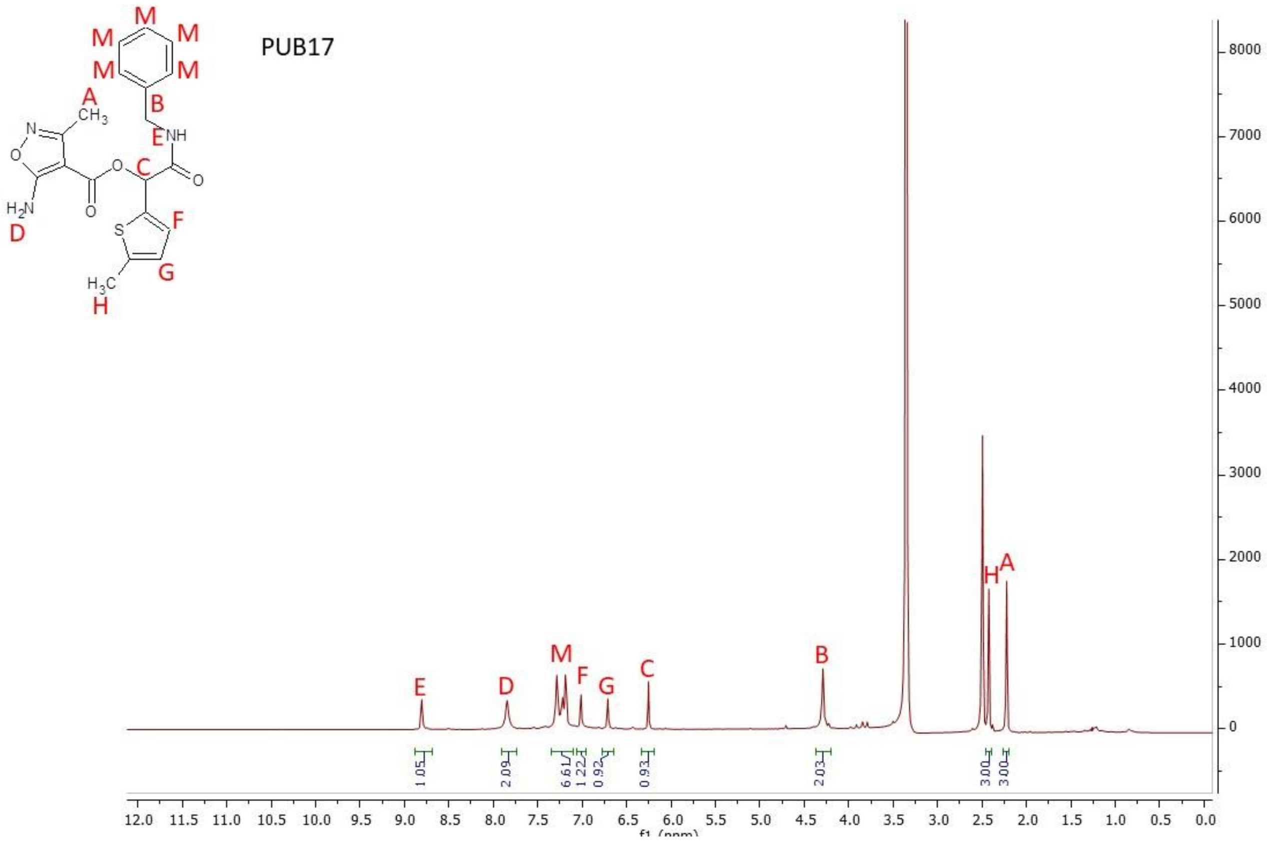

Figure S19. ^{1}H NMR spectrum of compound PUB17 in DMSO-d_6.

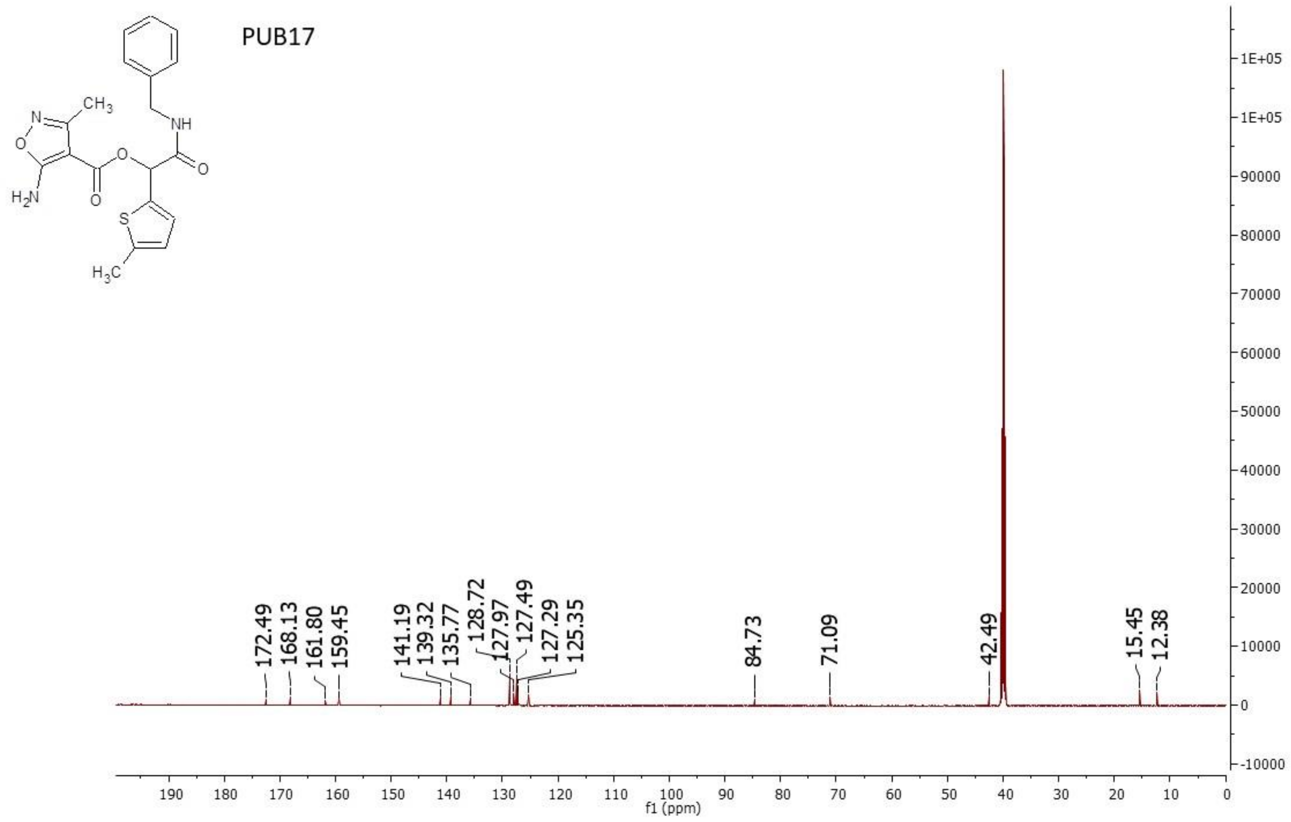

Figure S20. ^{13}C NMR spectrum of compound PUB17 in DMSO-d_6.

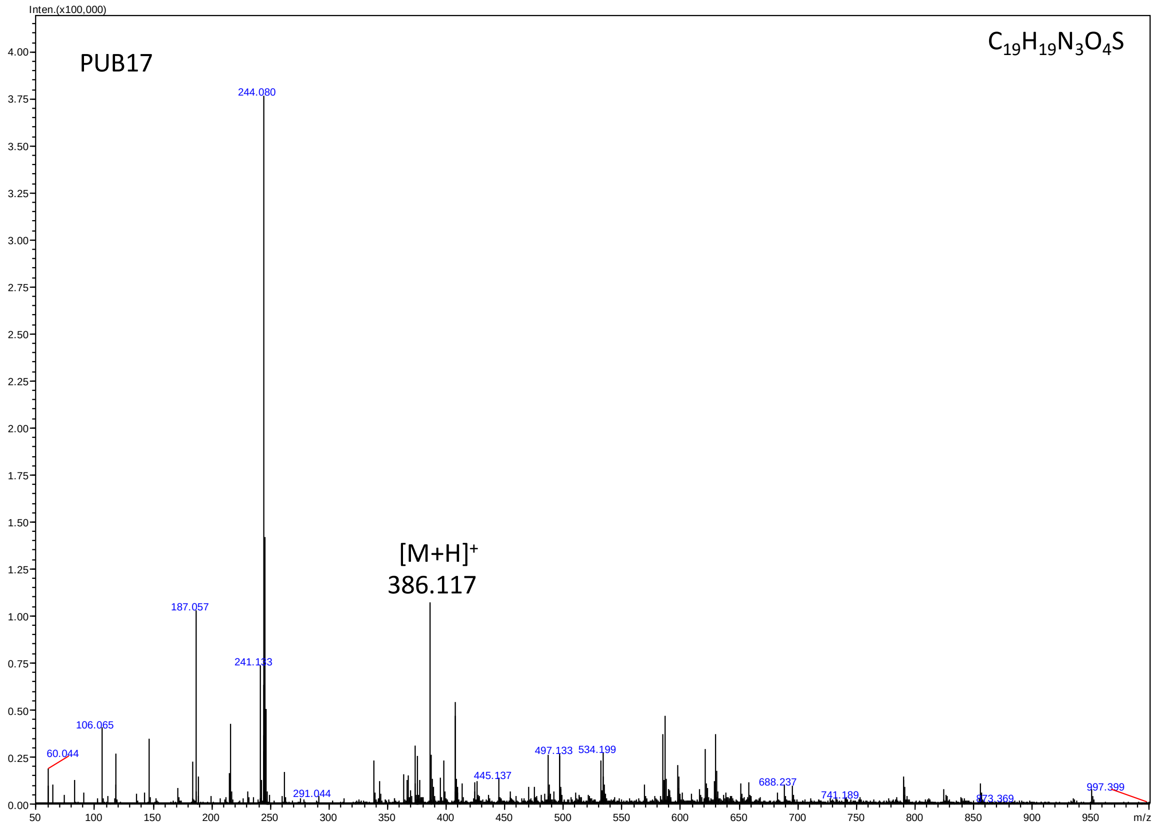

Figure S21. ESI-MS spectrum of compound PUB17.

1.8 Analysis of ^{1}H NMR, ^{13}C NMR and ESI-MS ^{1}H and ^{13}C NMR spectra of compound PUB18

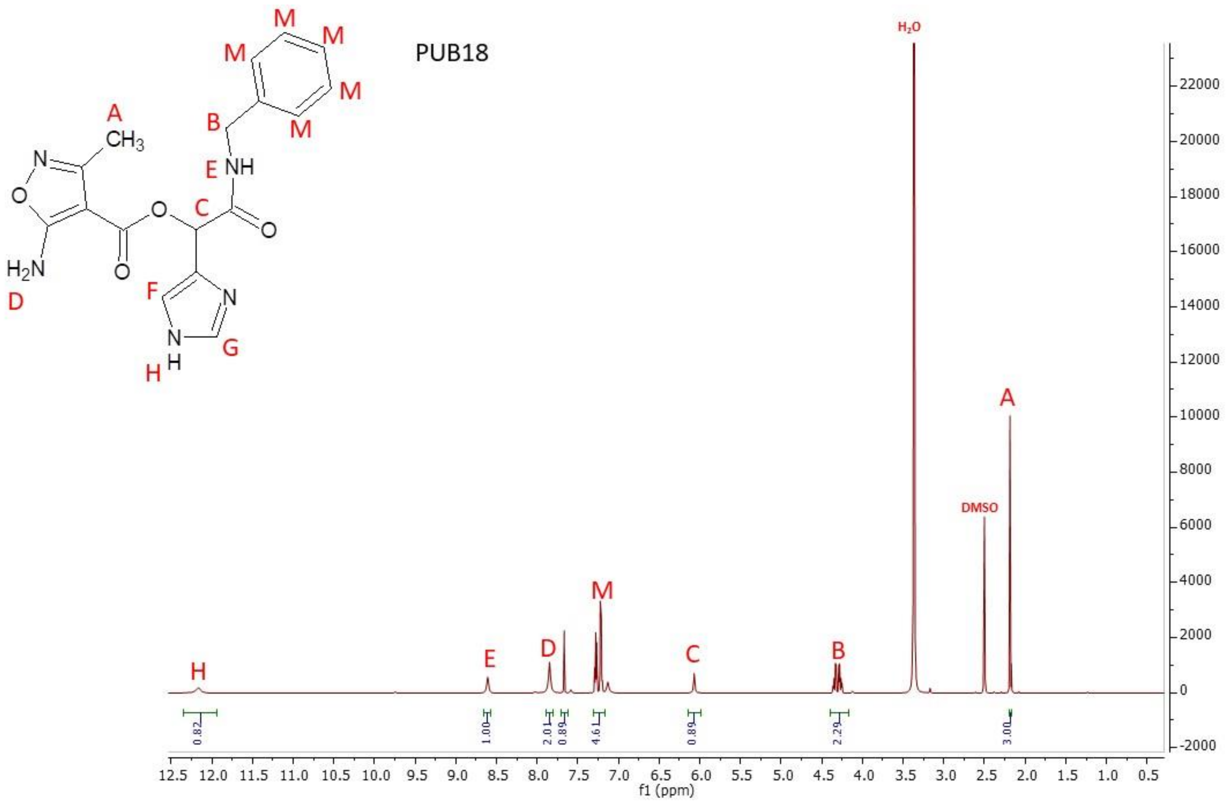

Figure S22. ^{1}H NMR spectrum of compound PUB18 in DMSO-d6.

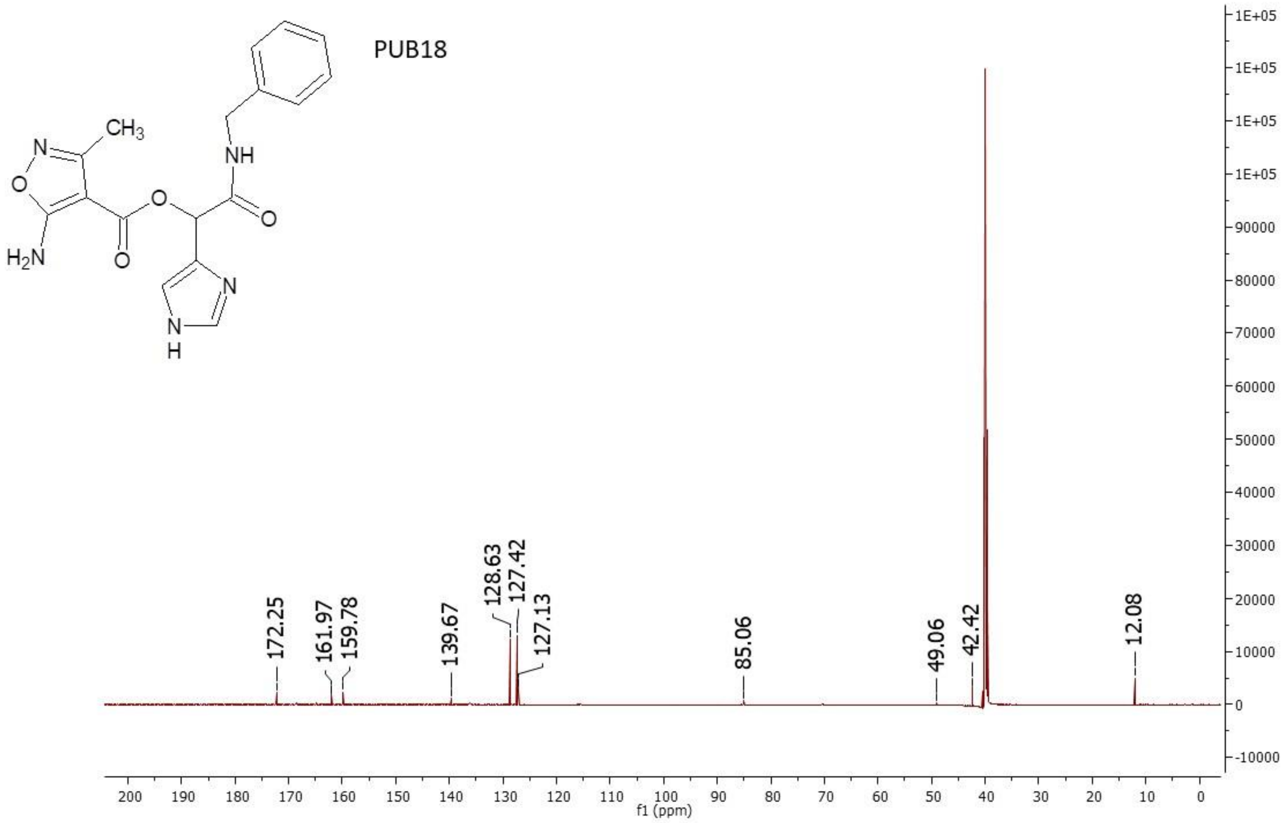

Figure S23. ^{13}C NMR spectrum of compound PUB18 in DMSO-d_6.

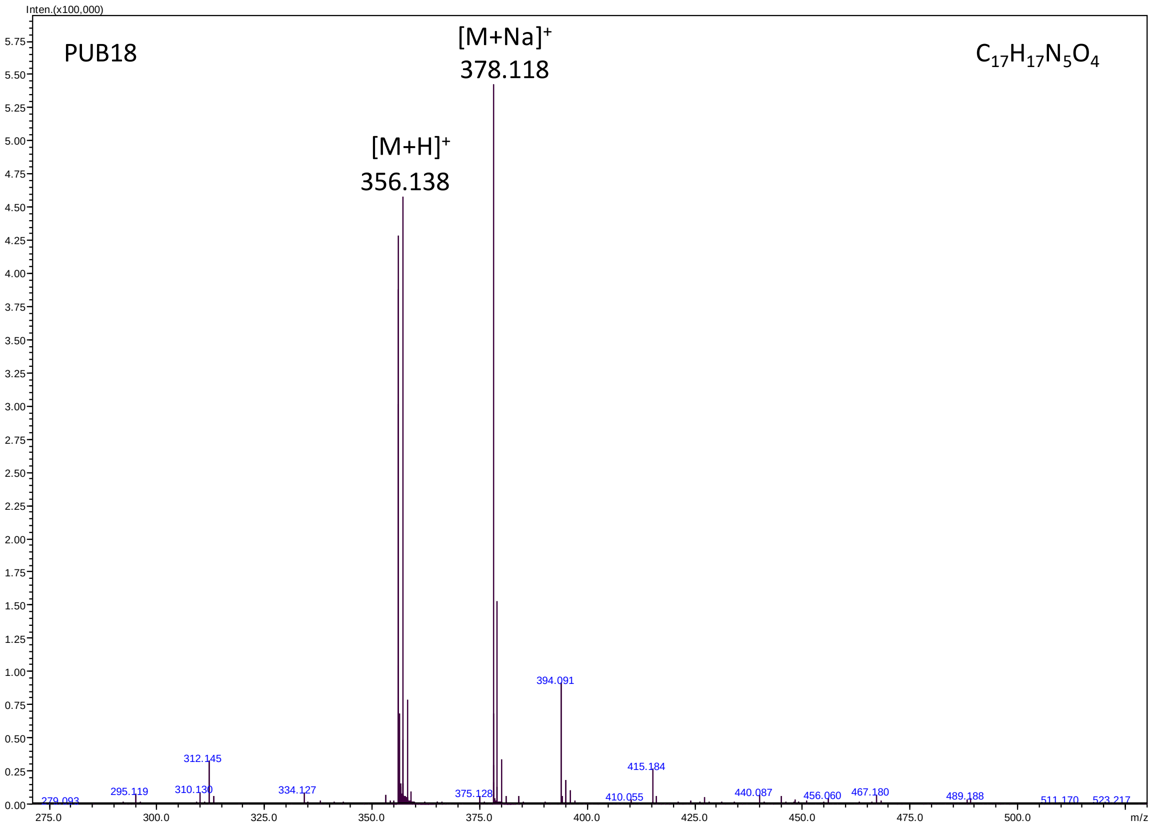

Figure S24. ESI-MS spectrum of compound PUB18.

2. HYSICOCHEMICAL PROPERTIES, PHARMACOKINETICS AND ADME ACTIVITY

Table 1. Physicochemical properties of compounds PUB11-PUB18

		Compound							
	Parameter	PUB11	PUB12	PUB13	PUB14	PUB15	PUB16	PUB17	PUB18
Lipinski's rules	MW (molecular weight) Optimal 100-600	442.335	450.314	364.427	372.406	439.537	377.466	385.445	355.354
	nHD (number of hydrogen bond donors) Optimal 0-7	2	7	2	2	2	2	2	3
	nHA (number of hydrogen bond acceptors) Optimal 0 - 12	7	2	8	8	7	7	7	7
	logP (Log of the octanol/water partition coefficient) Optimal 0-3	3.736	3.604	2.369	2.236	4.641	3.282	3.150	1.503
